# Fingolimod versus interferon beta 1-a: Benefit–harm assessment approach based on TRANSFORMS individual patient data

**DOI:** 10.1177/20552173221117784

**Published:** 2022-09-07

**Authors:** Alessandra Spanu, Hélène E Aschmann, Jürg Kesselring, Milo A Puhan

**Affiliations:** Epidemiology, Biostatistics and Prevention Institute, 27217 University of Zurich, Zurich, Switzerland; Epidemiology, Biostatistics and Prevention Institute, 27217 University of Zurich, Zurich, Switzerland; 166607 Department of Epidemiology and Biostatistics, University of California San Francisco, San Francisco, CA, USA; RehaKliniken Valens, Valens, Switzerland; Epidemiology, Biostatistics and Prevention Institute, 27217 University of Zurich, Zurich, Switzerland

**Keywords:** Benefit–harm assessment, multiple sclerosis, fingolimod, interferon beta-1a, health status, adverse events

## Abstract

**Background:**

Fingolimod is a disease-modifying drug approved for multiple sclerosis but its benefit–harm balance has never been assessed compared to other active treatments.

**Objectives:**

Our aim was to compare the benefits and harms of fingolimod with interferon beta-1a using individual patient data from TRial Assessing injectable interferon versus FTY720 Oral in RRMS trial.

**Methods:**

We modelled the health status of patients over time including Expanded Disability Status Scale measurements, relapses and any adverse events. We assessed the mean health status between arms and the proportion of patients whose health deteriorated or improved relatively to baseline, using a prespecified minimal important difference of 4.6. We performed sensitivity analyses to test our assumptions.

**Results:**

Main and sensitivity analyses favoured fingolimod 0.5 mg over interferon beta-1a. The average health status difference was 1.01 (95% CI 0.93–1.08). Patients on fingolimod 0.5 mg were 0.47 (95% CI: 0.35–0.63, *p* < 0.001) times less likely to experience a relevant decline in health status compared to interferon beta-1a patients, with a number needed to treat of 7.10 [5.18, 11.23].

**Conclusions:**

Fingolimod's net benefit over interferon beta-1a did not reach the clinical relevance over 1 year, but the decreased risk for health status deterioration may be more pronounced more long term and patients may prefer less treatment burden associated with fingolimod. 
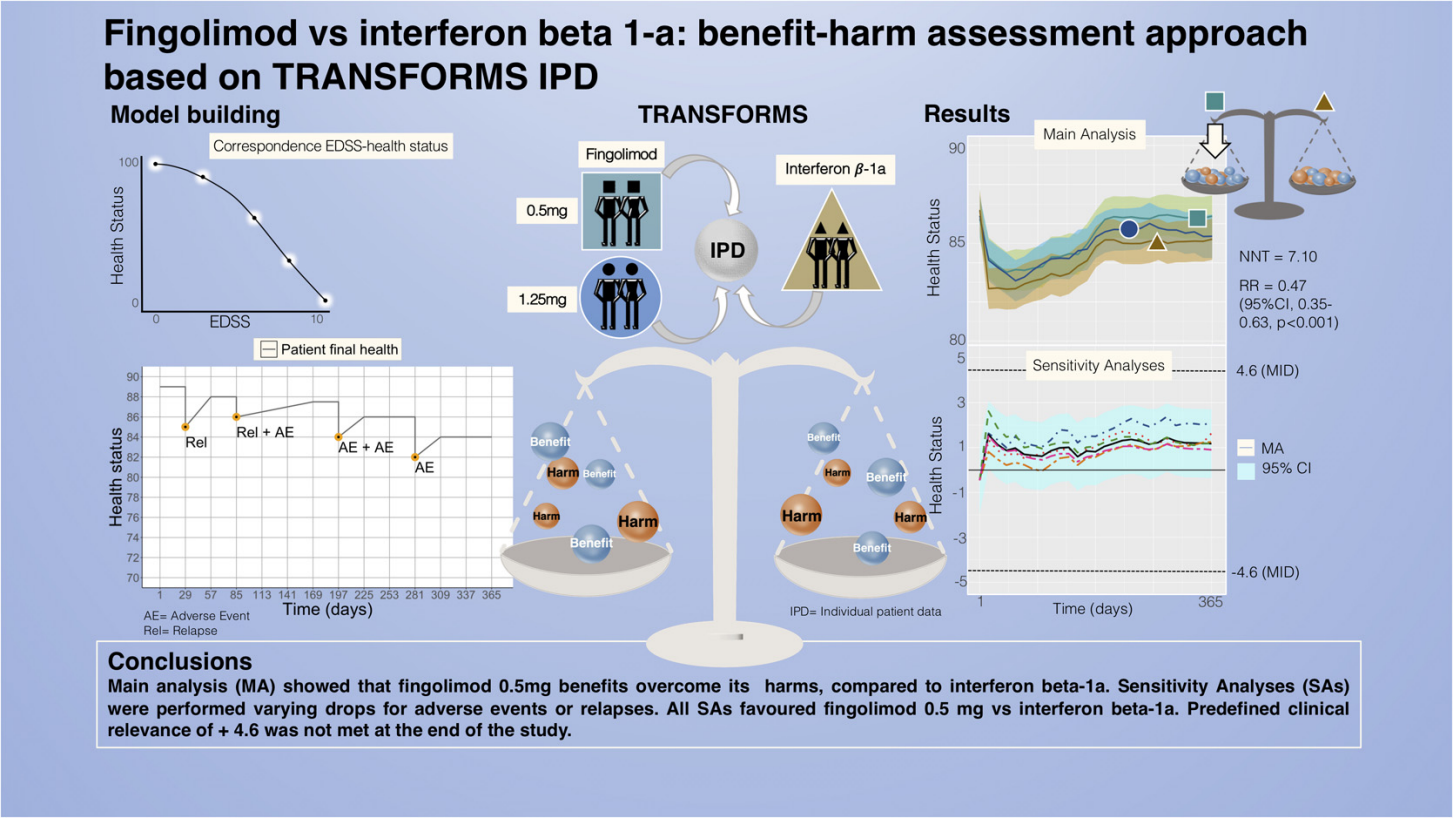

## Introduction

Fingolimod is a relatively new disease-modifying drug (DMD) for multiple sclerosis (MS) and its use is generally limited to more aggressive MS forms, since safety concerns about its use emerged post marketing.^1^ It is considered more effective, but less safe than interferon beta-1a, the first-line therapy established for many years. Fingolimod is approved, among other indications, for patients affected by relapsing-remitting multiple sclerosis (RRMS);^2^ however, its use differs by national recommendations.^3,4^

It is not straightforward to compare the balance of benefits and harms between treatments even if compared in a head-to-head trial. A quantitative benefit–harm assessment is helpful when there are multiple risks and benefits, with differing incidence and importance, and can provide useful, transparent evidence for guidelines recommendations and regulatory agencies.^5–7^ We found network meta-analyses of immunotherapies that compared benefits and harms but these were performed without incorporating a quantitative benefit–harm assessment in the analysis.^8,9^ A quantitative benefit–harm assessment study of fingolimod versus placebo was performed before,^10^ but to our knowledge, there is no such an analysis for the head-to-head comparison of fingolimod versus interferon beta-1a.

In June 2010, an advisory committee of the US FDA unanimously recommended the approval of fingolimod (previously called FTY720), recognized to provide substantial benefits in treating RRMS. The committee evaluated data from two studies, one comparing two different dosages of fingolimod versus placebo, the FREEDOMS study, and the second one comparing the same dosages of fingolimod versus interferon beta-1a, the TRANSFORMS study.

Our aim was therefore to assess the benefit–harm balance of fingolimod compared to interferon beta-1a, using individual patient data (IPD) from the TRial Assessing injectable interferon versus FTY720 Oral in RRMS (TRANSFORMS) study. We applied a quantitative method based on IPD, which, in comparison to aggregate data, has the advantage to include co-occurrence and sequence of events within the same patient. At the same time, the model based on IPD allows to incorporate follow-up assessments, relapses and any adverse events (AEs).

## Methods

### Study design

We designed a quantitative benefit–harm modelling study based on IPD of TRANSFORMS, a phase three, double-masked multicentre randomized trial, which compared two different dosages of fingolimod, 1.25 mg and 0.5 mg once daily (later referred to as fingolimod 1.25 mg and fingolimod 0.5 mg, respectively) to interferon beta-1a.^11^ The methods for the benefit–harm model have previously been described.^10^ The model estimated the health status of each participant of TRANSFORMS over a period of 1 year, by combining Expanded Disability Status Scale (EDSS) scores at baseline and scheduled visits, confirmed relapses and any AEs. After averaging the health status of participants in each arm, we compared the health status of the three arms of TRANSFORMS computing the difference in health status between curves.

Patients were eligible for TRANSFORMS if they had RRMS and were between 18 and 55 years of age, with an EDSS score between 0 and 5.5, experienced at least one relapse in the year prior to enrolment, or two or more relapses in the 2 years prior to enrolment. Patients previously treated for MS with a DMD such as interferons, glatiramer acetate, natalizumab and copaxone were also eligible.

We accessed TRANSFORMS IPD through the Clinical Study Data Request platform (online Supplemental material, section 5).

### Estimating the health status of individuals based on EDSS, relapses and AEs

To estimate the overall health status, we used a scale with values from 0 (worst possible outcome/death) to 100 (maximum health) as commonly used in health economic analyses or burden of disease studies;^12^ we then modelled the health of patients on this scale.

For each patient, we used the EDSS measurements collected at baseline and scheduled visits to estimate a health status baseline over the study period. Relapses and any AEs (online Supplemental material, Figure 4) are used to calculate drops in health status, as described before.^10^

For relapses, we considered severity (online Supplemental material, section 1.2.1), duration, hospitalization and/or corticosteroid therapy and type of recovery (full, partial and no recovery) for each patient. We incorporated only relapses confirmed by the EDSS in the model.^13,14^

We applied the same classification to AEs, which were coded using preferred terms of the Medical Dictionary for Regulatory Activities (MedDRA). As we described in the previously published model,^10^ we assigned a potential impact for each MedDRA code (very small, small, moderate and large) based on clinical judgment. Additionally, we included the symptom severity (no symptoms, mild, moderate and severe), duration and therapeutic intervention, to assign the drop in health status.

We performed two sensitivity analyses in which we considered extreme assumptions of drops in health status for relapses (very small/large) in comparison to the main analysis, to identify a range where the most probable health status would fall.

### Drops in health status due to relapses and AEs

We predefined how much the health status would drop following an event. We assumed a full drop on the first day of the event, then a gradual recovery starting the second day and considered it concluded at the day in which the event ended, according to what was reported in the datasets (online Supplemental material, sections 1.2.1 and 1.2.2). Ideally, preference surveys would have allowed us to empirically assign those drops,^15–18^ but we found no studies that covered such a wide collection of AEs. Tables 1 and 2 show our method for drop assignment.

**Table 1.** Drops in health status for relapses.

Outcome	Severity^a^	Treatment of relapse^b^	EDSS^c^ Rise^d^	Drop
**Relapse**	**Mild**	None	1.7	**9.1**
		Systemic corticosteroids	−	9.6
		Hospitalization	−	22.0
		SC^e^ + hospitalization	−	22.2
	**Moderate**	None	2.7	**15.8**
		Systemic corticosteroids	−	16.1
		Hospitalization	−	25.5
		SC + hospitalization	−	25.7
	**Severe**	None	3.5	**21.9**
		Systemic corticosteroids	−	22.1
		Hospitalization	−	29.7
		SC + hospitalization	−	29.8

^a^ These three severity categories are based on the Expanded Disability Status Scale.

^b^ Treatment of the relapse consists of four categories, where the fourth is the combination of the previous two (see online Supplemental material for a complete description of action taken combination).

^c^ EDSS: Expanded Disability Status Scale.

^d^ This column shows the EDSS score rise, which determines the drop in the health status compared to the stable status. EDSS scores are set according to relapse severity; each score is computed assuming the central value for severity range of values (mild (0–2), moderate (2.5 −3), severe (>3)) (11).

^e^ SC: Systemic corticosteroids.

**Table 2.** Adverse event (AE) categorizations and drops.

		Category of AEs according to potential impact on health status assigned for each MedDRA code			
Severity	Therapeutic action taken according to database	Very small impact	Small impact	Moderate impact	Large impact
No symptoms	None	1.9	2.5	3.7	5.0
	Study drug dose adjusted	2.1	2.7	3.9	5.1
	Minimal therapy given	3.5	3.9	4.8	5.8
	Moderate therapy given	8.2	8.4	8.8	9.4
	Study drug dose suspended	20.1	20.2	20.3	20.6
	(Prolonged) hospitalization	48.0	48.1	48.2	48.3
Mild symptoms	None	3.8	5.0	7.5	10.0
	Study drug dose adjusted	3.9	5.1	7.6	10.1
	Minimal therapy given	4.8	5.8	8.1	10.4
	Moderate therapy given	8.8	9.4	11.0	12.8
	Study drug dose suspended	20.3	20.6	21.4	22.4
	(Prolonged) hospitalization	48.1	48.2	48.6	49.0
Moderate symptoms	None	7.5	10.0	15.0	20.0
	Study drug dose adjusted	7.6	10.1	15.0	20.0
	Minimal therapy given	8.1	10.4	15.3	20.2
	Moderate therapy given	11.0	12.8	17.0	21.5
	Study drug dose suspended	21.4	22.4	25.0	28.3
	(Prolonged) hospitalization	48.6	49.0	50.3	52.0
Severe symptoms	None	15.0	20.0	30.0	40.0
	Study drug dose adjusted	15.0	20.0	30.0	40.0
	Minimal therapy given	15.3	20.2	30.2	40.1
	Moderate therapy given	17.0	21.5	31.0	40.8
	Study drug dose suspended	25.0	28.3	36.1	44.7
	(Prolonged) hospitalization	50.3	52.0	56.6	62.5

This table reports the drop value in health status associated with an adverse event, its severity and the therapeutic interventions taken. One adverse event can be treated with multiple interventions. Combinations of AEs are also possible and explained in online Supplemental material.

AEs are listed with the preferred MedDRA terms employed in the datasets, and separated into four categories. We evaluated the impact of AEs on health status under the supervision of clinical judgement. For example, headache was classified as an AE with very small impact, influenza-like illness as an AE with small impact, viral infection as an AE with moderate impact, macular oedema and seizures as AEs with large impact on health status.

MedDRA: Medical Dictionary for Regulatory Activities.

We built the drops for AEs by combining an algorithm where we defined the drops for superior terms, with a manual change of single AEs for preferred terms. This process could have led to small discrepancies within preferred terms; this is, however, non-differential for treatment arms using the same AEs coding across arms. We confirmed this in sensitivity analyses where we changed AEs drops and found no significant differences in comparison to the main results. We considered that several AEs could happen at the same time in our model,^19^ as well as that relapses could overlap with AEs.

We designed two different sensitivity analyses for AEs: in the first analysis, we modified the drop increase associated with event severity; in the second analysis, we excluded mild events to evaluate the impact on health status provided by moderate and severe AEs alone on the benefit–harm balance.

We performed a sensitivity analysis using different assumptions to convert EDSS scores in the health status, and set a cut-off for worst possible health/death at an EDSS value of 9, instead of 10. When we changed the drops for relapses or AEs, we kept the other model parameters constant.

### Censoring

At 1 year of TRANSFORMS study, 12% of patients with fingolimod 1.25 mg and 11% circa with interferon beta-1a, and 7% of patients with fingolimod 0.5 mg were censored. We used multiple imputation (25 imputed datasets) with predictive mean matching^20^ performed with the package mice in R^21^ to address censoring. We considered age, gender, arm, BMI and health status to predict the missing health status entries.

### Statistical analysis

We established a priori a minimal important difference (MID) of ± 4.6 to assess the clinical relevance to the health difference. We computed the MID as half the SD of the health status at baseline.^22^ Based on a systematic review,^23^ the anchor-based approaches analysed yielded more conservative values. We decided on a distribution-based approach for two reasons: it was not possible to identify any surveys that included all our outcomes, and in a study using an anchor-based approach the MID was set at 1 on the EDSS range of 0–5.5,^24^ which is identical to the range of baseline EDSS in TRANSFORMS. Considering the non-linearity of the EDSS conversion in health status scale values, a change of 1 on the EDSS corresponds to a MID of about 8 on the health status scale, which is larger than the distribution-based MID of 4.6. After a careful evaluation of these conditions, the distribution-based approach had the advantage of being computed directly on our sample, and using a MID of 8 would not have changed our main conclusions.

We computed the health status difference between groups over time, considering that if the lower limit of 95% confidence interval exceeds the MID of 4.6, the average health gain would have been of clinical relevance. In addition, we computed the proportion of patients who experienced a relevant improvement or decline compared to their baseline health status after 1 year of study; we then calculated the risk ratio for a relevant improvement or decline and the number needed to treat (NNT).

All analyses were performed using R version 3.4.3 on the Clinical Study Data Request (CSDR) platform.

### Data availability

The data that support the findings of this study are publicly available on the CSDR platform at request from the study sponsor Novartis.

## Results

### Characteristics of trial participants

TRANSFORMS enrolled a total of 1292 participants with a mean EDSS of 2.21 (SD 1.30), corresponding to a health status at baseline of 86.4 (SD 9.2) points, and a mean age of 36.1 years (SD 8.5). In total 870 (67.3%) were female. Participant characteristics were balanced across trial arms. A summary of the cohort baseline characteristics is shown in table 3.

**Table 3.** Summary of cohort baseline characteristics.

	Fingolimod 0.5 mg	Fingolimod 1.25 mg	Interferon beta-1a	Total
*Patient No.*	431	426	435	1292
*Discontinued*	33	57	49	139
*Death*	0	2	0	2
*Study time*	−	−	−	1 year
*Sex*				
*Female %*	65.4	68.8	67.8	67.3
*Age*				
*Mean (SD)*	35.8 (8.8)	36.7 (8.4)	36.0 (8.3)	36.1 (8.5)
*Health Status*				
*Mean (SD)*	86.2 (9.4)	86.4 (9.5)	86.7 (8.7)	86.4 (9.2)
*EDSS*				
*Mean (SD)*	2.24 (1.33)	2.21 (1.31)	2.19 (1.26)	2.21 (1.30)

### Difference in health status between fingolimod 0.5 mg and interferon beta-1a

**Figure 1.**
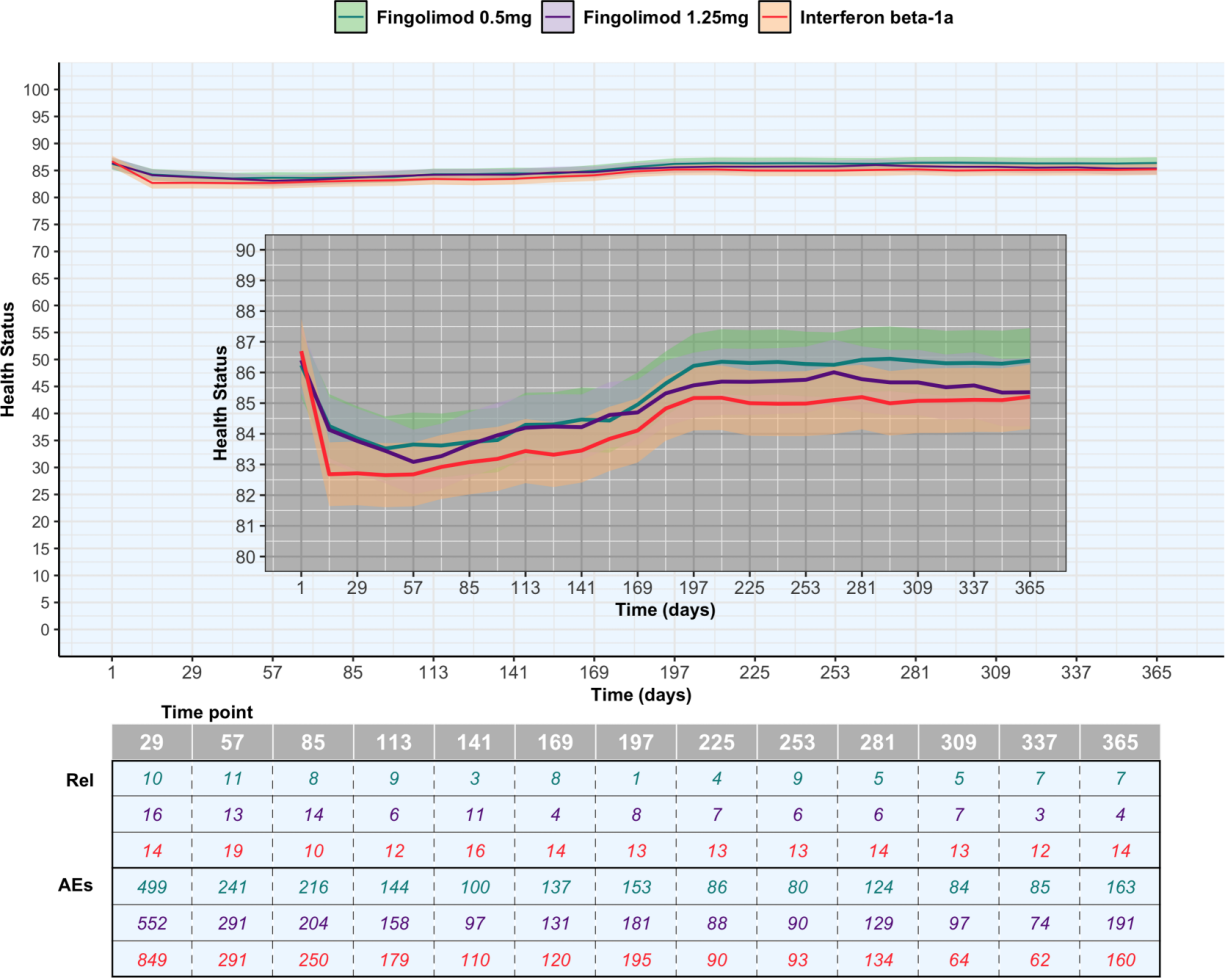
Mean health status of patients with fingolimod 0.5 mg, fingolimod 1.25mg and interferon beta-1a over 1 year.

Figure 1 shows the health status mean difference in patients on all three arms. Here, we focus on the difference between fingolimod 0.5 mg and interferon beta-1a (for results on fingolimod 1.25 mg, see online Supplemental material). The 1-year mean difference was + 1.01 (95% CI 0.85 to 1.15), which did not reach the MID of 4.6 points (for results on fingolimod 1.25mg vs. interferon beta-1a, see online Supplemental material). The table in Figure 1 reports all AEs and relapses that occurred during the study by trial arm over time. AEs occurred most frequently at the beginning of the study.

The figure shows the mean and 95% confidence interval of the mean health status in each group.

The larger plot represents a full health status scale (0–100); the figure inside shows a partial health status scale (*y*-axis, from 80 to 90) and health status curves for the three treatment groups; below two tables show the total relapse numbers during the corresponding time periods, covering 28 days each (*x*-axis) and the total adverse event (AE) numbers during the same corresponding time points. The table font colors report the top centre legend for the three arms: Green – fingolimod 0.5 mg; purple – fingolimod 1.25mg; and red– interferon beta-1a.

To investigate a potential impact of double counting both, EDSS changes and relapses, we performed a sensitivity analysis that only considered the EDSS at baseline but not at follow-up visits. We found results that are consistent with the main analysis (see online Supplemental Material, section 7).

**Figure 2.**
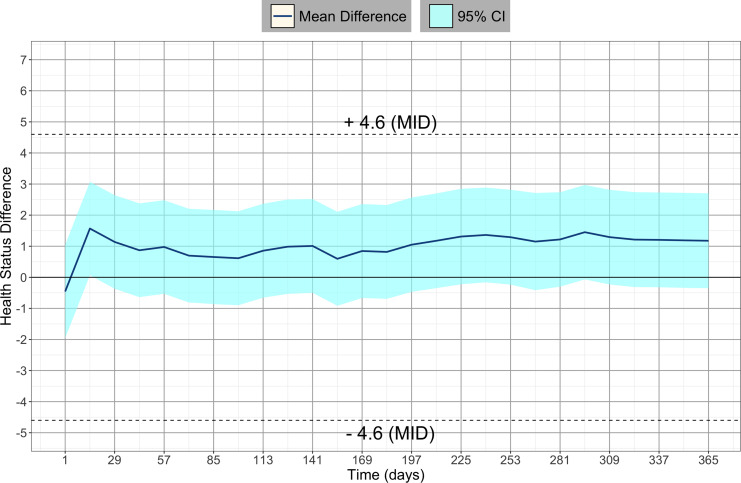
Difference in mean health status between patients with fingolimod 0.5 mg and interferon beta-1a in the TRANSFORMS trial. If the difference is positive, patients on fingolimod 0.5 mg had a better health status on average than those on interferon beta-1a ± 4.6.

Using imputed data, we found that 12.4% (53/429) in the fingolimod 0.5 mg group and 26.5% (114/431) in the interferon beta-1a group experienced a relevant decline in health status (i.e. MID lower than −4.6 points). This corresponds to a relative risk of 0.47 (95% CI: 0.35−0.63, *p* < 0.001) and a NNT of 7.1 [5.2, 11.2] to prevent one relevant decline in health status. We obtained a consistent result when performing the same analysis without imputation (discarding withdrawn patients) (Figure 2).

MID: minimal important difference.

### Sensitivity analyses with large and small drops for relapses

**Figure 3.**
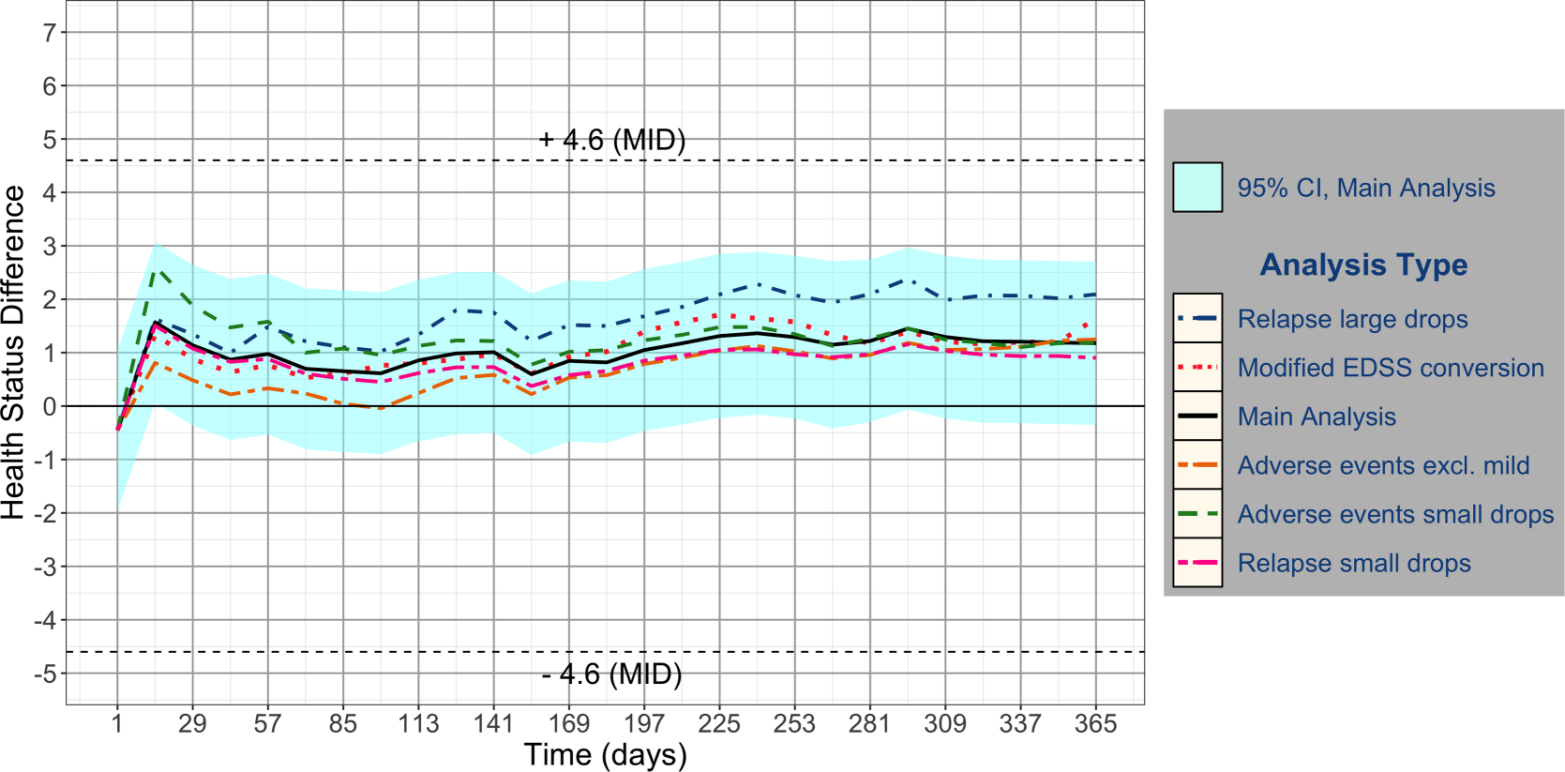
Sensitivity analyses. Figure 3 shows all sensitivity analyses compared with the main analysis mean difference.

Figure 3 shows the results of all sensitivity analyses. The mean difference for the sensitivity analysis with small drops for relapses (Figure 3, *Relapse small drops*) favoured fingolimod 0.5 mg, with a health status difference of 0.80 (95% CI 0.74−0.87). The sensitivity analysis with large drops for relapses (Figure 3, *Relapse large drops*) displays the most favourable health status difference of 1.63 (95% CI 1.52−1.74). Both sensitivity analyses gave similar results as the main analysis, with mean differences in the health status well below the MID.

The error band represents the 95% CI of main analysis mean difference.

Adverse events excl. mild: sensitivity analysis excluding mild adverse events; EDSS: Expanded Disability Status Scale; MID: minimal important difference.

As shown with FREEDOMS analyses, the benefit–harm balance of fingolimod is only moderately sensitive to changes for relapse and AE drops.

### Sensitivity analyses with different drops for AEs

When we changed drops for AEs as shown in Figure 3, with one analysis where drops increased less with severity (Figure 3, *AEs small drops*), and the second analysis that considered AEs except mild events (Figure 3, *AEs excl. mild*) we found similar differences in health status between the trial arms as in the main analysis (1.23, 95% CI: 1.13−1.31 and 0.66, 95% CI: 0.58−0.78, respectively).

### Sensitivity analysis with different conversions of EDSS to health status scale

In one sensitivity analysis, we changed the assumptions for EDSS conversion^25^ (Figure 3, *Modified EDSS conversion*), and set the worst possible health or death to an EDSS of 9.^26^ The average difference in health status was 1.06 (95% CI: 0.97−1.14). This example shows how easily the EDSS conversion can be adjusted with this method, allowing for a great flexibility even when testing extreme assumptions as we did in this analysis. Indeed, the results are in agreement with those in the main analysis.

## Discussion

Our analysis showed that the health status average followed the same behaviour in all arms, with a health status around 83 to 86, declining at the beginning of the study, then recovering in the first 6 months, and stabilizing afterwards. This range of health status for patients affected by MS is reasonable since the average health status in the general population at a similar age (35–45 years) is a bit higher with a mean EQ-5D score of 0.89 on a scale with a maximum of 1.^27^

Fingolimod arms started to separate after 6 months. By 1 year, patients with fingolimod 0.5 mg reached the best health status in comparison to patients in the other arms on average, although not with statistical significance. Interferon beta-1a showed the worst progression in comparison to the other two arms, with higher number of relapses and AEs over the entire study period. The health status curve in this arm decreased steeply at the beginning of the study in comparison to the other treatment groups, mainly due to high rates of AEs in the beginning, then it reverted and stabilized similarly to the other arms in the second half of the year. Modelled health status of fingolimod 1.25 mg was between that of interferon beta-1a and fingolimod 0.5 mg, with more relapses and AEs over the study duration, in comparison to fingolimod 0.5 mg.

Neither the main nor any sensitivity analyses showed a clinically relevant difference over 1 year in the average health status between patients treated with fingolimod or interferon beta-1a.

### Clinical importance

Our results showed that there was a modest net benefit for fingolimod 0.5 mg that did not reach clinical relevance over 1 year, which implies that fingolimod 0.5 mg was clinically equivalent to interferon beta-1a. In our main analysis, the average difference between fingolimod 0.5 mg and interferon beta-1a was very small and both curves stabilized below the MID for the entire study duration. Since the MID value for clinical significance was not met, we further investigated the proportion of patients with a significant health deterioration in both groups, obtaining an NNT of 7.1 to prevent one relevant decline in health status over 1 year of treatment.

The MID value gives a threshold above which one treatment is significantly better than its comparator. Fingolimod 0.5 mg and interferon beta-1a are used in the management of MS as effective drugs to slow disease progression, to slow the accumulation of disabilities and to reduce the number and duration of exacerbations.^28,29^ When considering classical primary outcomes in MS – time to progression in disability, annualized relapse rate, time to relapse – and AEs *separately*, fingolimod 0.5 mg performs better than interferon beta-1a.^11,30^ In our model, the benefits and harms are accounted for in the same metric, and each outcome receives a weight depending on severity, duration and type of event. Our results suggest that the slight advantage in health status offered by fingolimod 0.5 mg over 1 year may be negligible at a population level; however, this difference may be still important for individuals, depending on how relapses, type of AEs and disease progression are perceived. In addition, some patients may favour fingolimod because the treatment burden is generally perceived to be lower with orally taken drugs whens compared to injections. We did not include treatment burden in our analysis since such information was not available from the trial’s IPD data.

Concerning AEs, the majority were more frequent in the first quarter of the year in both drugs, with a peak in the first month. In our main analysis, interferon beta-1a had the highest number of AEs, fingolimod 0.5 mg the lowest; yet this difference played a small role in the results. In the interferon beta-1a arm, AEs were more abundant but mostly mild and/or had a short duration, which is consistent with previous study findings and post marketing signalling (FDA Adverse Events Reporting System – FAERS – Public Dashboard).^31^ Among mild AEs, ‘headache’ was almost twice as common with fingolimod 0.5 mg intake than interferon beta-1a; ‘macular oedema’, a severe AE reported with the use of fingolimod 0.5 mg,^30^ was observed in two patients under fingolimod 0.5 mg treatment, and one patient under interferon beta-1a treatment. During TRANSFORMS there were two deaths caused by disseminated viral infections, both in the fingolimod 1.25 mg arm. One of them happened at the beginning of the extension phase. Progressive multifocal leukoencephalopathy, a rare brain infection associated with fingolimod 0.5 mg and other DMDs,^32^ was not observed in TRANSFORMS.

### Preference sensitivity of benefit–harm balance

As observed in our benefit–harm assessment based on the FREEDOMS study,^10^ the model was very stable; even extreme assumptions, such as that one shown in the analysis with different EDSS conversions nor large drops for relapses, did not meaningfully change the results. With respect to modelling assumptions concerning AEs, we showed that assigning small or large drops did not impact the results. Our primary analysis emphasizes small differences in average health status between treatment groups. This analysis does not consider possible differences in treatment burden that, as explained above, likely further favour fingolimod. Moreover, our secondary analysis indicates that with fingolimod 0.5 mg, a risk of a relevant decline over 1 year is approximately half of that with interferon beta-1a. This less conservative approach could imply that the difference may still be clinically relevant to some. Especially considering variation in patient preferences, individuals could have varying willingness to accept relapses or specific AEs^33^ potentially changing the benefit–harm balance. In this context, patients’ preferences should play a role in the decision-making about the available therapies.

### Strength of the study

We based our model on IPD, which allowed us to monitor the evolution of the health status of each patient over time and discover possible clusters of events. We considered all AEs irrespective of how the relationship with the study drug was assessed by the clinician (suspected / not suspected). This approach allowed us to avoid potential bias in the adjudication. Our model based on IPD allowed a more precise estimation of the benefit–harm balance than if we had compared mere counts of events.

### Limitations of the study

When considering the AEs computed at each time point, we purposely included only AEs with confirmed severity but not around a thousand events with unknown severity, so we may have slightly overestimated the overall health status for patients of all three treatment arms. One year of study duration is not sufficient to identify rare AEs such as progressive multifocal leukoencephalopathy, and differences in the efficacy preventing relapses and disease progression may have become more important after 1 year. We could not perform subgroup analyses due to the small sample size of the study. Similarly as in FREEDOMS, the frequency of AEs declined over time in all arms. This phenomenon could be due to over-reporting of AEs at the beginning of the study, to a tolerance-induced effect, or underreporting towards the end of the study. Lower rates of AEs after a few months could mean that a longer follow-up time might impact the benefit–harm balance.

Finally, TRANSFORMS was powered for detecting a difference in annual relapse rates, and was not designed for our benefit–harm model. A larger sample size may have shown a statistically significant (although still not clinically relevant) improvement of estimated health status with fingolimod. A longer time frame may have favoured fingolimod more strongly. Clinical trials are not typically designed with a focus on the benefit–harm balance.^34^ Our model can serve as a case study to explore how MS drug trials could be analysed and reported with more focus on the benefit–harm balance.

## Conclusions

To our knowledge, we performed the first quantitative benefit–harm assessment comparing interferon beta-1a and fingolimod 0.5 mg, using a model that we designed to evaluate MS DMDs.

Our analyses show that fingolimod 0.5 is clinically equivalent to interferon beta-1a over 1 year when all side effects and relapses are considered modelling their severity and temporal structure. This analysis adds to our previous results comparing fingolimod to placebo,^10^ where fingolimod was favoured over placebo. Our results strengthen the growing evidence that it would be beneficial to incorporate quantitative benefit–harm metrics at the level of RCTs.

It is worth emphasizing that the relatively large value of the MID is a consequence of the heterogeneous value of the health status of patients at baseline. Therefore, our approach to determine the MID, while common, could be over conservative.

As we observed in our analysis of FREEDOMS IPD,^10^ our method for benefit–harm assessment appears as a useful tool to compare MS DMDs, in particular when the balance of benefits and harms is challenging to determine.

## Supplemental Material

sj-pdf-1-mso-10.1177_20552173221117784 - Supplemental material for Fingolimod versus interferon beta 1-a: Benefit–harm assessment approach based on TRANSFORMS individual patient data Click here for additional data file. Supplemental material, sj-pdf-1-mso-10.1177_20552173221117784 for Fingolimod versus interferon beta 1-a: Benefit–harm assessment approach based on TRANSFORMS individual patient data by Alessandra Spanu, Hélène E Aschmann, Jürg Kesselring and Milo A Puhan in Multiple Sclerosis Journal – Experimental, Translational and Clinical
