# Supplementary material for: Fingolimod versus interferon beta 1-a: Benefit–harm assessment approach based on TRANSFORMS individual patient data
Source: Mult Scler J Exp Transl Clin. 2022 Sep 7;8(3):20552173221117784. doi: 10.1177/20552173221117784 (PMC9459487; doi:10.1177/20552173221117784)
Supplement: sj-pdf-1-mso-10.1177_20552173221117784 - Supplemental material for Fingolimod versus interferon beta 1-a: Benefit–harm assessment approach based on TRANSFORMS individual patient data [file sj-pdf-1-mso-10.1177_20552173221117784.pdf]

SUPPLEMENTAL DATA

SECTION 1. DETAILED METHODS

Section 1.1 Conversion of EDSS scores to estimated health status

We used three determinants to estimate the health status over time: the EDSS (expanded disability status scale) (9), relapses and adverse events.

In a first step, we used the EDSS measured at baseline and every three months to estimate the health status. These were scheduled visits, where patients were in a stable state (i.e. free of relapse for at least four weeks). We converted EDSS scores to the health status scale (0 to 100) based on the literature as follows: we extracted the Global Burden of Disease (GBD) 2015 disability weights for mild (0.183), moderate (0.463) and severe (0.719) MS (10). The corresponding EDSS categories are 0 -3.5 for mild MS, 4 -6.5 for moderate and above 7 for severe MS (12). Accordingly, we assumed that GBD disability weights of 0, 0.183, 0.463, 0.719 and 1 corresponded to EDSS scores of 0, 3, 6, 8, 10, respectively. We converted GBD disability weights to health status on a scale from 0 to 100, as it is usual in cost-effectiveness analyses (figure 4), using the formula:

Health status = (1-GBD disability weight) * 100.

We then fitted a spline to convert EDSS scores to the health status scale. For example, as a result, an EDSS score of 2.5 corresponded to a health status of 85.6, an EDSS score of 5 to a health status of 64.5, and an EDSS score of 7 to a health status of 41. In a second step, we added the effect of relapses and adverse events on the initial estimate of health status for each individual in the trial (estimated based on EDSS).

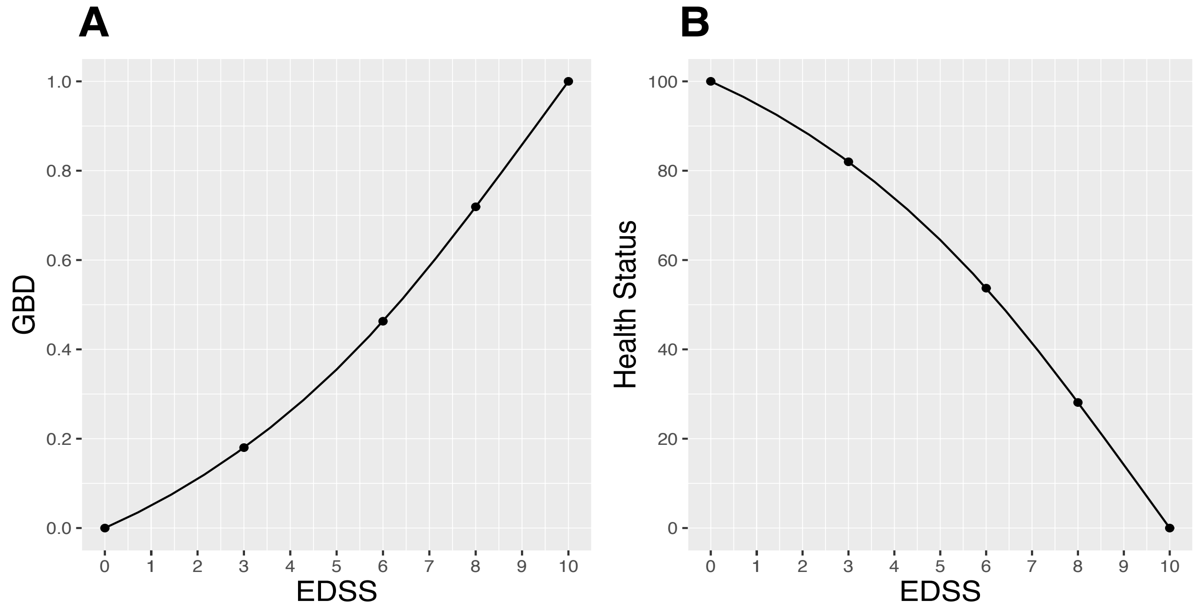

Figure S1. Scale Conversion from EDSS to health status, based on disability weights from the Global Burden of Disease study

Panel A. Conversion function of EDSS scores (0, 3, 6, 8, 10) to disability weights (0, 0.18, 0.463, 0.719, 1) from the Global Burden of Disease study.

Panel B. After re-scaling of disability weights to a health status scale (100, 82.0, 53.7, 28.1, 0), this shows the function we used to convert EDSS scores to an initial health status.

GBD = Global Burden of Disease;

EDSS = Expanded Disability Status Scale.

Section 1.2 Impact on adverse events and relapses on estimated health status

Section 1.2.1 Relapse Drops

We combined relapse severity and treatment of the relapse (steroid therapy and/or hospitalisation) to define the initial drop in health status on the day of the event. We defined three different trajectories of recovery (described below) depending on whether the patient had a full, partial or no recovery after a relapse event.

The initial drop due to a relapse was defined by the relapse severity and the action taken:

$$Drop = \sum_i \sqrt{w_{severity}^2 + w_{action\ i}^2}$$

where $w_{severity}$ is the weight we defined for each severity of the relapse (value of 9 for mild, 15 for moderate and 22 for severe relapses), and $w_{action\ i}$ is the weight we defined for the action taken (value of 3 for steroid therapy given and 20 for a hospitalization). For instance, for a patient with a mild relapse who required no additional therapy, we assigned a drop equal to 9.0 points on the health status scale. Or, if a patient experienced a severe relapse that required hospitalisation and treatment with steroids, we assigned a drop of 29.9 (=$\sqrt{22^2 + 20^2 + 3^2}$). Thus, we accounted the possibility that these two actions could happen together, by combining them as described in the formula above.

We implemented in the model a variable that defined the recovery level from relapse: complete, partial and no recovery. This variable was accounted in the computation with the following schema: for no recovery, we subtracted the computed drop from the health scale from the time of the event until censoring or the end of the study For partial recovery we assumed that half of the initial drop persisted after the recovery time in the health scale; for total recovery, the drop reached the null value on the last day of event, meaning that the health score prior the event was regained when the relapse was over. Also for partial and no recovery we considered that the health status reached at the end of a relapse lasted until censoring or the end of the study. A new event (new relapse, new adverse event) always changed the reached health status; adverse events normally changed it temporarily, relapses could change it permanently.

The model considered relapses that occurred only during the core phase of the trial and that were confirmed by EDSS measurements.

We incorporated only relapses confirmed by the EDSS in the model. Unfortunately, the dataset did not report the scores of such measurements, so that we had to attribute them a corresponding EDSS score based on relapse clinical assessment and literature (12,13). Datasets classified relapses in mild, moderate, severe, very severe; if a relapse was coded as mild, the EDSS rise compared to the stable status was estimated at 1.7 (the central value for the range, see Table 1, main text) and the corresponding health status drop was 9.1, assuming no treatment. In the TRANSFORMS study, patients did not experience very severe relapses.

Section 1.2.2 Adverse Events

We accounted in our model the type of adverse event (AE), the duration of the event and assumed a progressive recovery until the last day of event (i.e. the day of full recovery). We took into account all AEs that patients experienced during the study.

We grouped AEs in four categories based on their impact on health status. The impact was very small, small, moderate or large (Table 2). We assigned each AE to a category based on classifications found in literature and consensus, when possible. We classified all the other weights comparing AE severity with these AEs, keeping a consistent evaluation while assigning an AE to his category.

The TRANSFORMS datasets included variables that defined AE typology (name or preferred term according to MedDRA definition, 937 in total, see Table 6), AE severity and therapeutic actions taken due to an AE. TRANSFORMS classified severity in 4 levels: no symptoms, mild, moderate, severe. Six levels of actions were defined in TRANSFORMS, which described the action taken due to an event. In the main analysis, we assumed an exponential function for severity and action taken, giving a drop according to variable level. All variables combined together to give the final formula:

$$\sqrt{\sum_{i,j} (AE_i^2 + AT_j^2)}$$

In this formula, AE represents the weight category plus AE severity, AT is the action taken subsequent the AE.

In the first sensitivity analysis, we considered that severity grew linearly; in the second one we simply took off mild AEs to get only the impact of severe AEs (see main text for full sensitivity analysis descriptions).

We excluded “Multiple sclerosis relapse” and “Multiple Sclerosis” as AEs to avoid double counting of events. Using the same rationale, we decided to not incorporate in our analysis Multiple Sclerosis Functional Composite (MSFC) assessments, since it could lead to double counting of the functional status.

Section 1.2.3 Co-occurring events

If many AEs happened at the same time, they were added in quadrature according to the above formula (17).

Section 1.2.4. Health Status Example

In the figure below we presented an example of individual health behaviour, starting from EDSS measurements taken at scheduled visits. The health status at baseline is 2. The patient is in a stable condition (free of relapses for at least four weeks), and his health status fluctuates over the study period respect to the baseline.

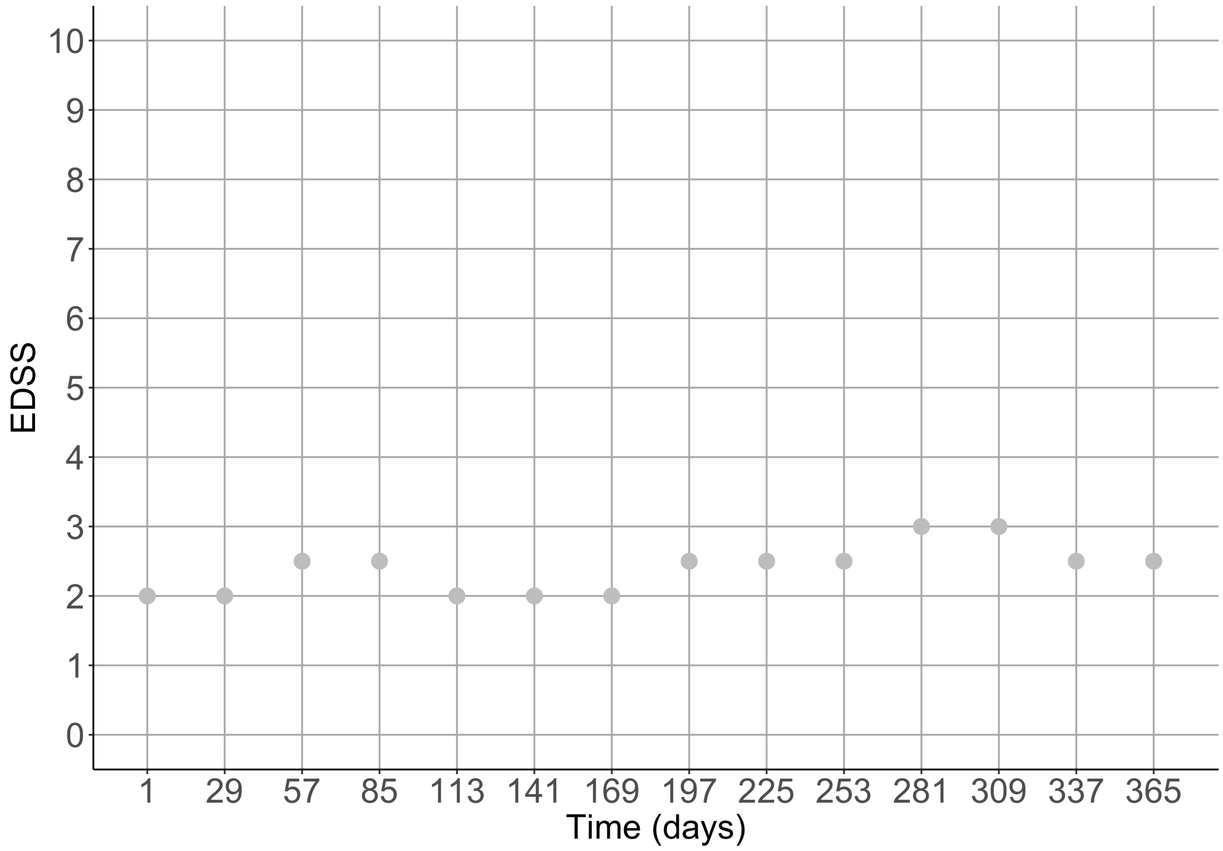

Figure S2. Example of EDSS measurements at baseline and scheduled visits

Patient EDSS scores fluctuate in time, with intervals of worsening health alternated by recoveries.

All EDSS scores are then converted in health status scores, as described in section 1.1. Patient health status corresponds to 89 points at baseline.

If a relapse or an AE happened within 3 months after randomization, the patient experienced a temporary drop in the health status. In this example the health dropped down to 83 (i.e. assuming a drop in health status of ~6 points based on a priori determined rules), for a number of days or weeks (as documented in the database).
Other events occurred (i.e. AEs, any type), lowering temporarily the health status even more as shown in figure 7, where multiple events occurred bringing the health status at 78 points at the first half of the first year. At the beginning of the second year the health recovers and falls again, stabilising at ~86 points in the second year towards the end of the study.

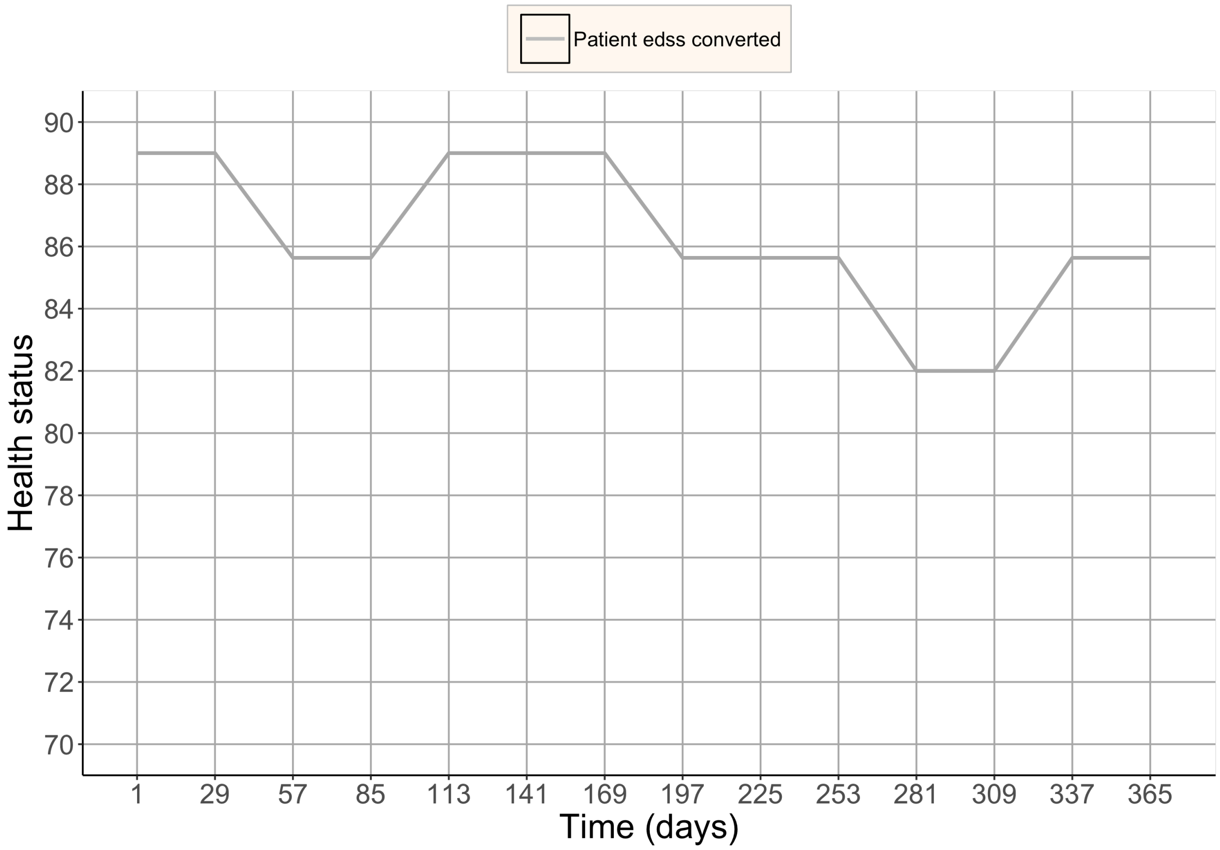

Figure S3. Initial estimate of the individual health status

Patient EDSS scores are converted in health status points with the method described in Section 1.1. The figure shows an initial estimate of the patient health based solely on EDSS scores. All scores are extracted for each patient in each TRANSFORMS arms.

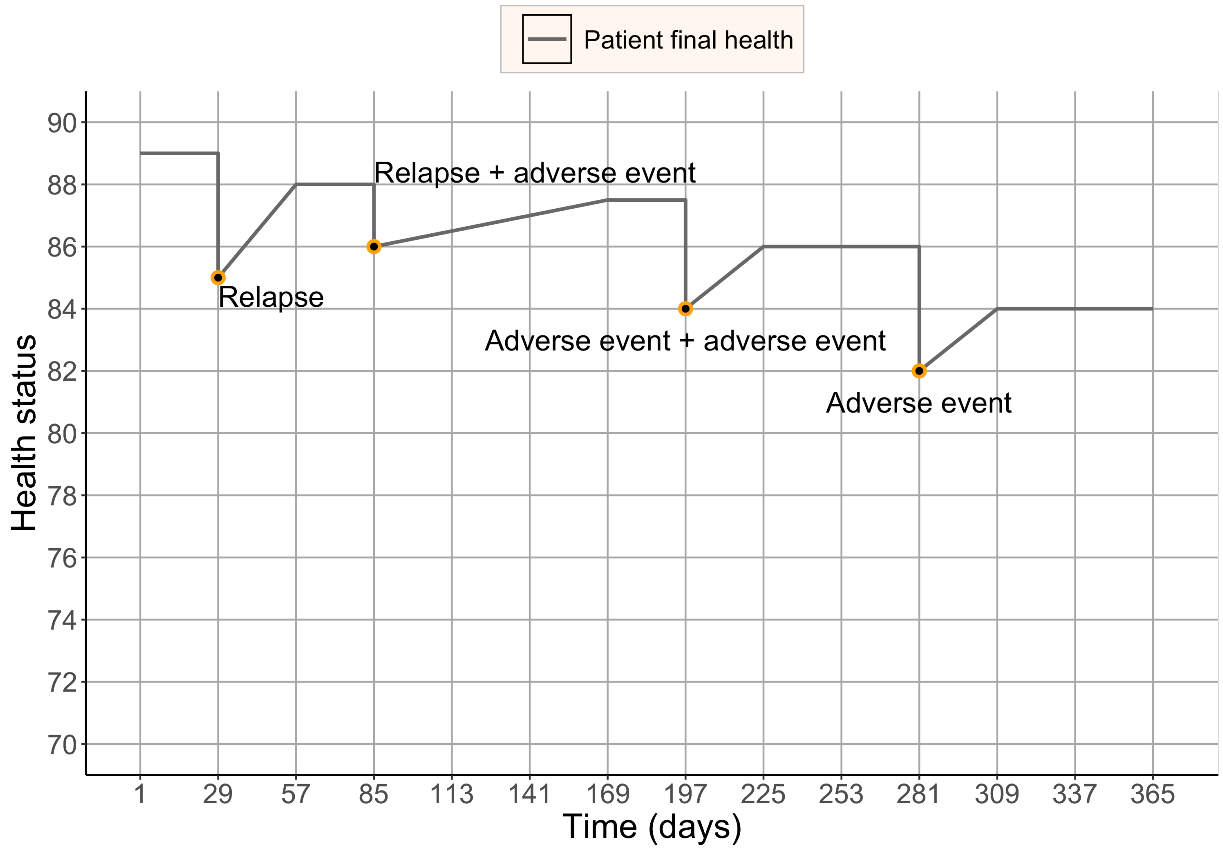

Figure S4. Combination of EDSS, relapses and adverse events gives the final health
Variations downwards (drops) are determined by a sudden appearance of an adverse event or relapse, followed by recovery.

The recovery process starts the second day of the event and is progressive until the end of the event.

Duration of all events are reported in the datasets and implemented in the analysis. The model allows for reaching the maximum health status, which is 100, as shown in the example. Measurement points are extracted every 14 days. The final health is represented by the dotted line, which accounts for EDSS scores, relapses and adverse events. Criteria for relapse and adverse event conversion are described above in section 1, Detailed Methods. See also table 1 and table 2 in the main text. These are simulated data used to illustrate the method.

Section 1.4. Sensitivity analyses with different drops for relapses and adverse events

Table 3 shows the drops used in sensitivity analysis *Relapse small drops* (figure 3).

Table 3. Small drops for relapses

Outcome	Severity[1]	Treatment of relapse[2]	EDSS [3]	Drop
**Relapse**	**Mild**	none	1	**5**
		Systemic corticosteroids	-	5.8
		Hospitalization	-	20.6
		SC[4] + hospitalization	-	20.8
	**Moderate**	none	2	**11**
		Systemic corticosteroids	-	11.4
		Hospitalization		22.8
		SC + hospitalization	-	23.0
	**Severe**	None	**3**	**18**
		Systemic corticosteroids		28.3
		Hospitalization		26.9
		SC + hospitalization	-	27.0

[1]Three categories, drops converted from Expanded Disability Status Scale values;

[2]Four categories, the latter being the combination of the previous two.

[3] EDSS scores are set according to very mild relapse severity;

[4] Systemic corticosteroids.

Table 4 shows the drops used in sensitivity analysis *Large Drops for Relapses* (figure 3)

Table 4. Large drops for relapses

Outcome	Severity[1]	Treatment of relapse[2]	EDSS [3]	Drop
**Relapse**	**Mild**	None	3.5	**21.9**
		Systemic corticosteroids	-	22.1
		Hospitalization	-	29.7
		SC[4] + hospitalization	-	29.8
	**Moderate**	None	5	**35.5**
		Systemic corticosteroids	-	35.6
		Hospitalization	-	40.8
		SC + hospitalization	-	40.9
	**Severe**	None	**6.5**	**52.3**
		Systemic corticosteroids	-	52.4
		Hospitalization	-	56.0
		SC + hospitalization	-	56.0

[1]Three categories, drops converted from Expanded Disability Status Scale values;

[2]Four categories, the latter being the combination of the previous two.

[3] EDSS scores are set according to relapse severity; the score was determined considering extremely high values for severity;

[4] Systemic corticosteroids.

Table 5 shows the drops used in sensitivity analysis *Adverse events small drops* (figure 3)

Table 5 Drops in health status for adverse events, linear growth

**Severity of symptoms**	**Therapeutic action taken according to database**	**Category of adverse events according to potential impact on health status**			
		**Very small impact**	**Small impact**	**Moderate impact**	**Large impact**
No symptoms	None	0	0	0	0
	Study drug dose adjusted	2	2	2	2
	Minimal therapy given	2	2	2	2
	Moderate therapy given	5	5	5	5
	Study drug dose suspended	10	10	10	10
	(Prolonged) hospitalization	20	20	20	20
Mild symptoms	None	7.5	10	15	20
	Study drug dose adjusted	9.5	12	17	22
	Minimal therapy given	9.5	12	17	22
	Moderate therapy given	12.5	15	20	25
	Study drug dose suspended	17.5	20	25	30
	(Prolonged) hospitalization	27.5	30	35	40
Moderate symptoms	None	11.25	15	22.5	30
	Study drug dose adjusted	13.25	17	24.5	32
	Minimal therapy given	13.25	17	24.5	32
	Moderate therapy given	16.25	20	27.5	35
	Study drug dose suspended	21.25	25	32.5	40
	(Prolonged) hospitalization	31.25	35	42.5	50
Severe symptoms	None	15	20	30	40
	Study drug dose adjusted	17	22	32	42

Minimal therapy given	17	22	32	42
Moderate therapy given	20	25	35	45
Study drug dose suspended	25	30	40	50
(Prolonged) hospitalization	35	40	50	60

This table lists the drops in health status for one adverse event and its severity and therapeutic consequence. Drops for combinations of adverse event are explained in the Supplemental Data, section 4.3.

[1]Adverse events (preferred MedDRA terms) were separated into 4 categories based on clinical judgement about the impact the adverse event is likely to have on health status when mild and no action is taken. For example, nausea was classified as an adverse event with very small impact, bradycardia as an adverse event with small impact, viral bronchitis as an adverse event with moderate impact, and macular oedema as an adverse event with large impact on health status (see all AEs, table e-1, section 3). These drops present a linear growth for severity.

SECTION 2. SENSITIVITY ANALYSIS WITH MODIFIED EDSS CONVERSION

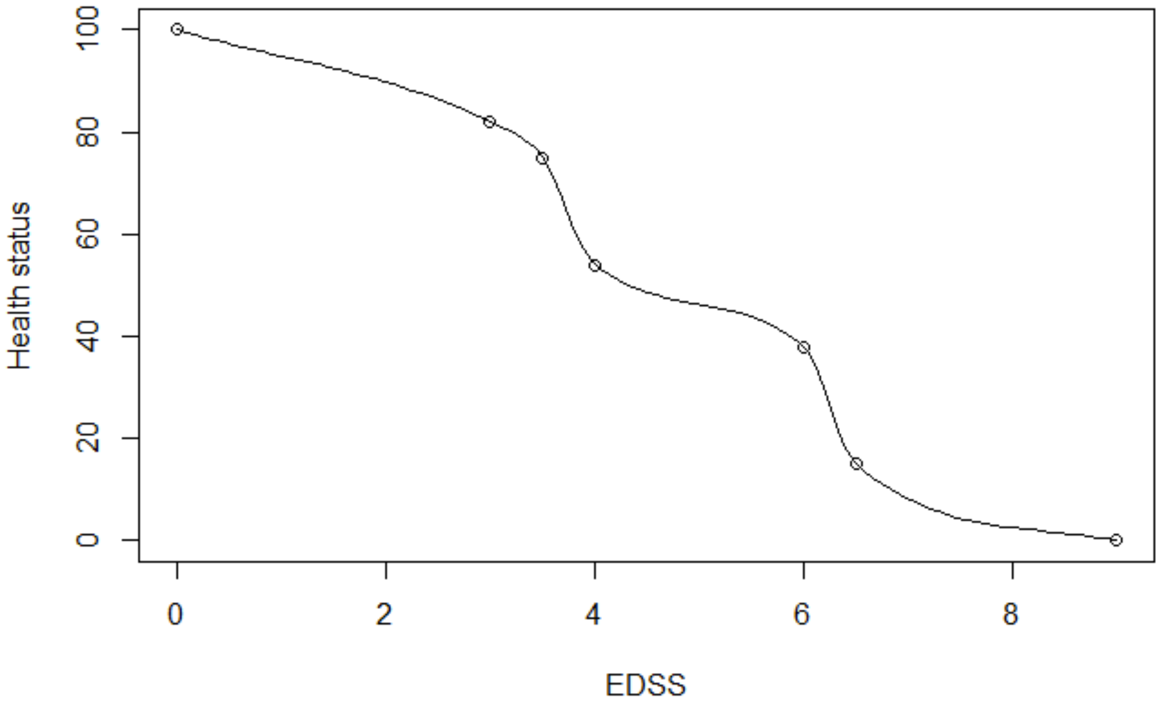

Figure S5. Modified EDSS conversion used to build up a sensitivity analysis

It shows the relationship between EDSS and health status. EDSS transitions are extremely different, with the major impact concentrated within 3-4 and 5-6 scale steps. Worse health/death is set at 9. Results of this sensitivity analyses are presented and discussed in the manuscript.

SECTION 3. NUMBER OF RELAPSES AND PREVIOUS DISEASE MODIFYING THERAPIES INTAKE PER PATIENT

In this separate analysis, we evaluated the proportion of patients who were taking MS medications before entering the study and developed relapses during the first three months of the study. To note: this analysis is highly sensitive to the type of variables selected.

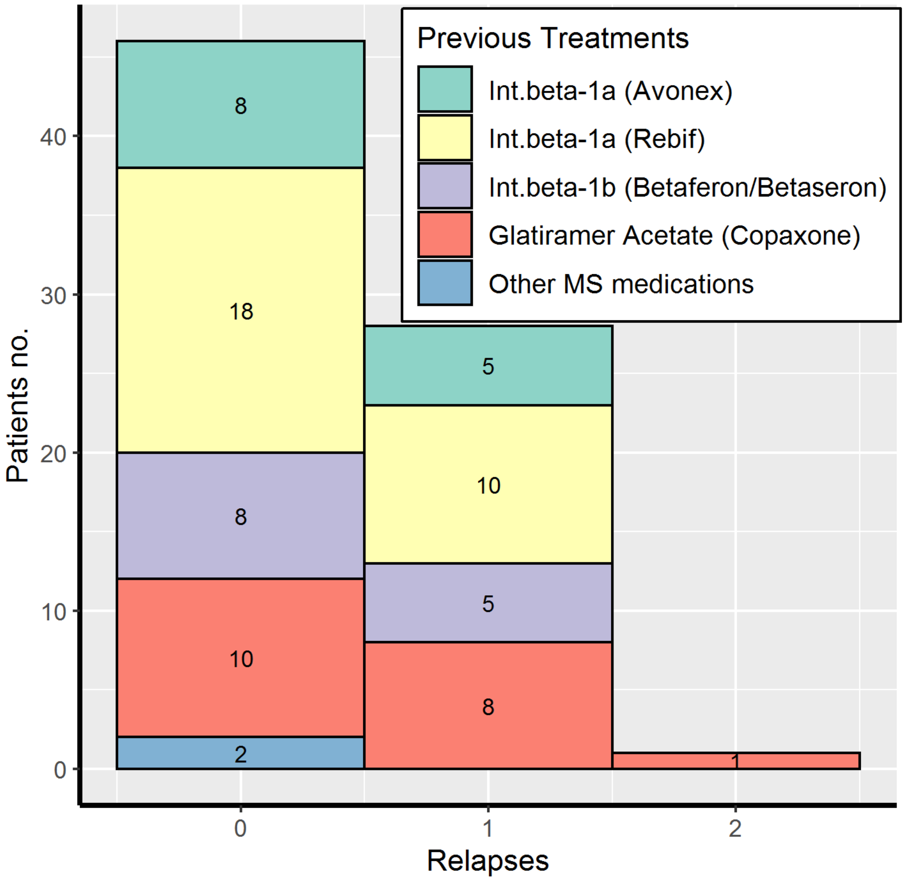

Figure S6. Number of relapses by previous treatment in the fingolimod 0.5mg trial arm

In this analysis we looked at the association between previous treatments, study arm and number of relapses during the first three months of TRANSFORMS trial. The rationale for this analysis was to explore a possible relationship between type of drug intake and number of relapses experienced. Patients enrolled in fingolimod 0.5mg experienced less relapses during this period (total number of relapses per arm, total relapses per patient, which were never more than 1).

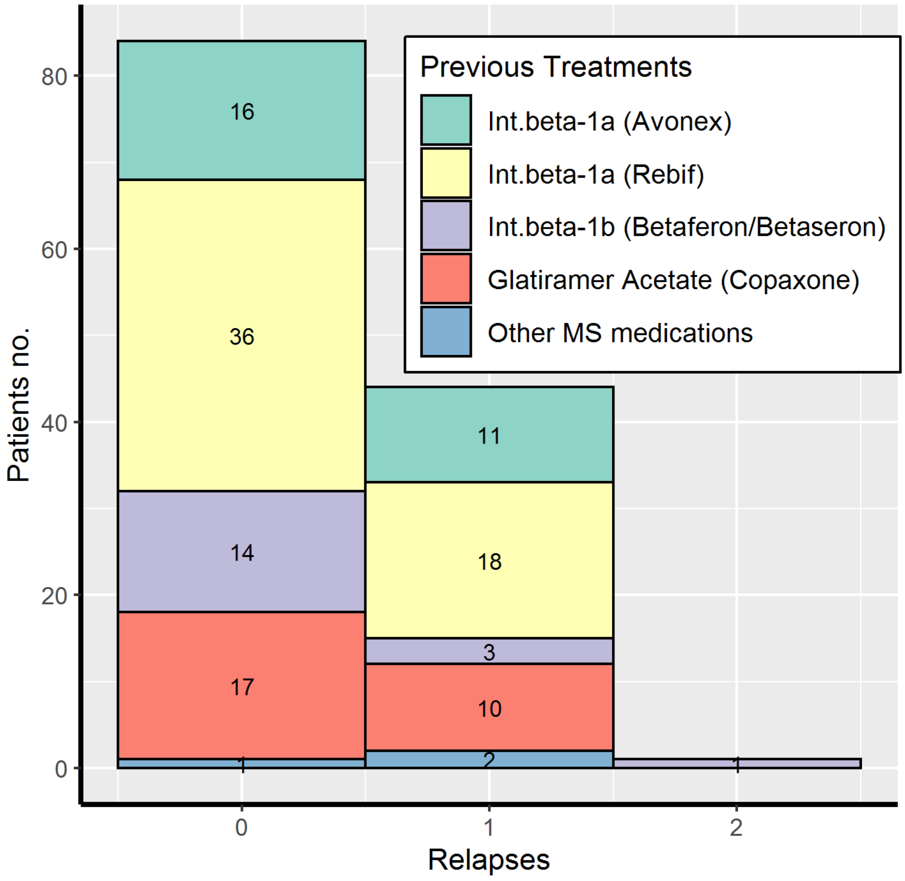

Figure S7. Number of relapses by previous treatment in the interferon beta-1a arm

This figure shows the association between previous treatments, interferon beta-1a arm and number of relapses during the first three months of TRANSFORMS trial. Patients enrolled in interferon beta-1a arm experienced more relapses during this period when compared to the other two arms (total number of relapses per arm, total relapses per patient, which could reach 2 per patient).

To note: the results of this analysis may change depending on the type of variables selected.

SECTION 4. RESULTS FOR FINGOLIMOD 1.25 mg

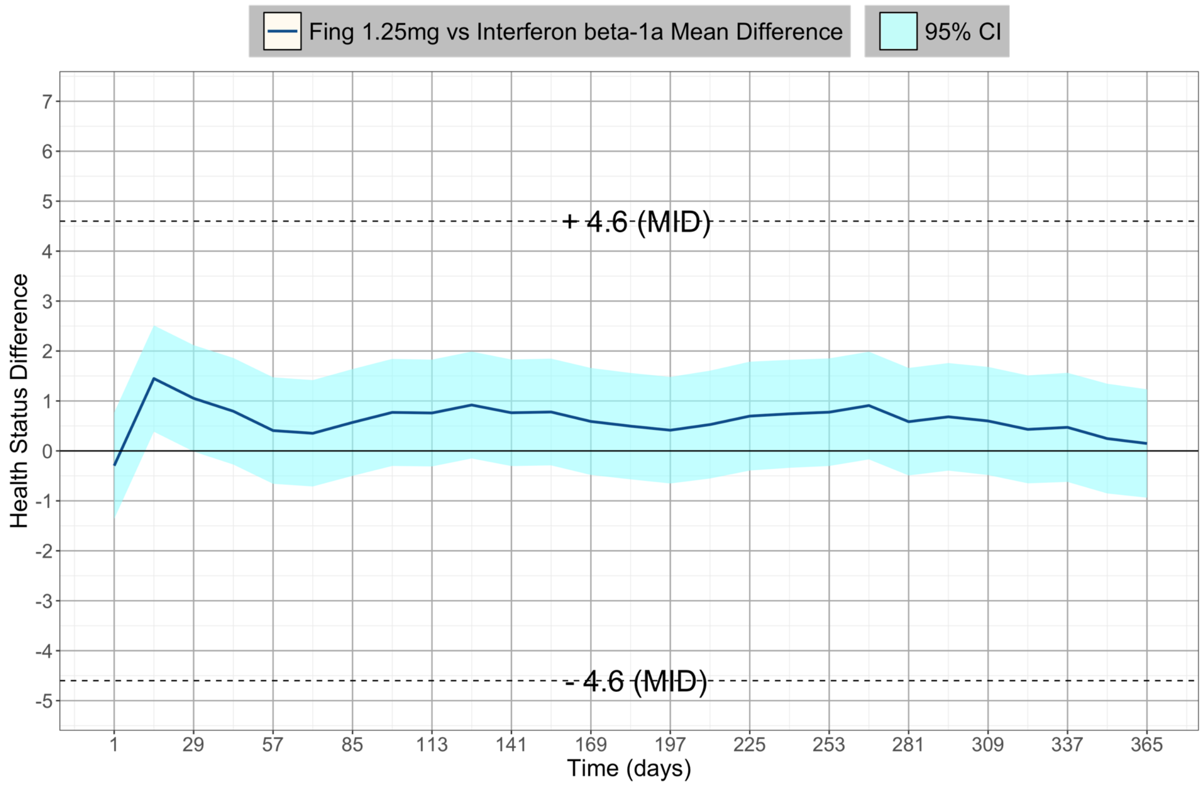

Figure S8. Difference in mean health status between patients with fingolimod 1.25mg and Interferon beta-1a in the TRANSFORMS study

The difference is positive, therefore patients on fingolimod 1.25 had a better health status on average than those on interferon beta-1a.

± 4.6 : Minimal Important Difference (MID).

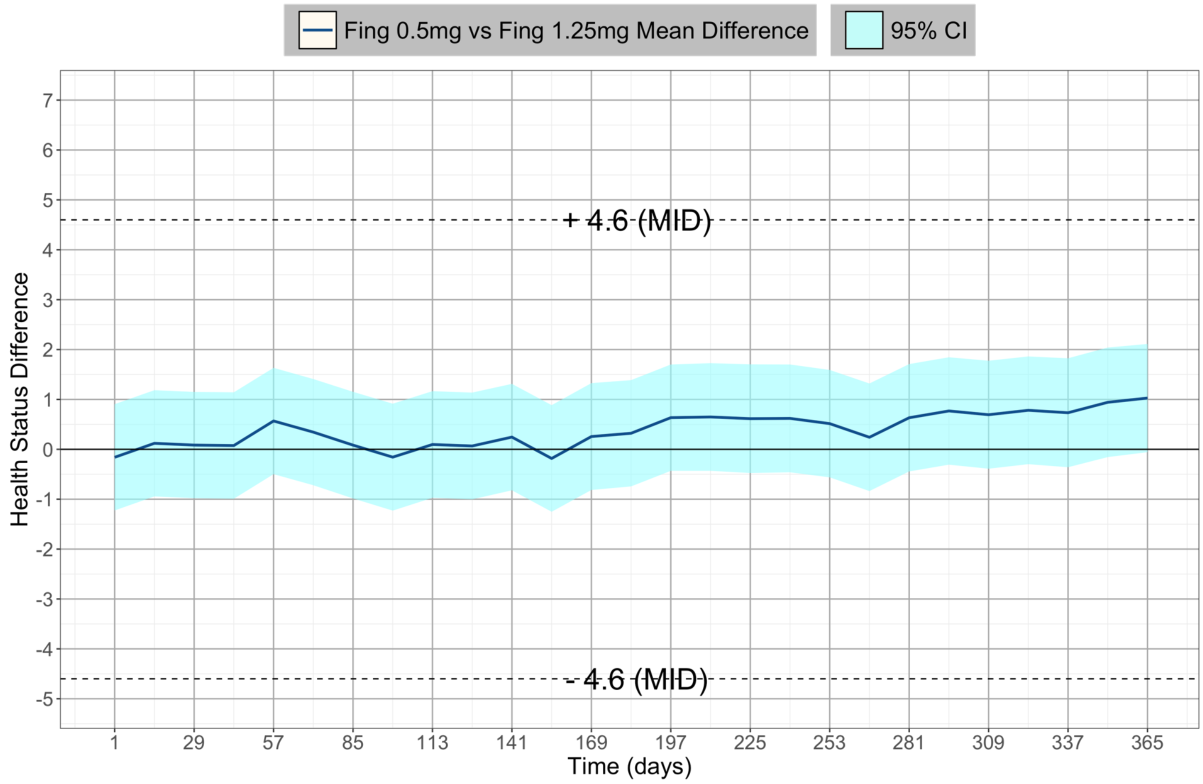

Figure S9. Difference in mean health status between patients with fingolimod 0.5mg and fingolimod 1.25mg in the TRANSFORMS study

The difference is positive, therefore patients on fingolimod 1.25 arm had a worse health status on average than those on fingolimod 0.5mg. This difference is not statistically significant.

± 4.6 : Minimal Important Difference (MID).

SECTION 5. DATA ACCESS

We accessed TRANSFORMS IPD through the Clinical Study Data Request platform (CSDR, ClinicalStudyDataRequest.com) which is a consortium where sponsors share clinical trial data for research purposes. All applications that aim to receive deposited data have to be approved by an Independent Research Board. A data sharing agreement (DSA) is signed by the study sponsor and the research group. In our case, the study sponsor Novartis gave us access to the data after signing the DSA (1933). The research group is completely free and independent from the sponsor on the study question, development of the methods, the analysis and the interpretation of the results, writing of the manuscript or the decision to publish.

SECTION 6. EDSS VARIATION

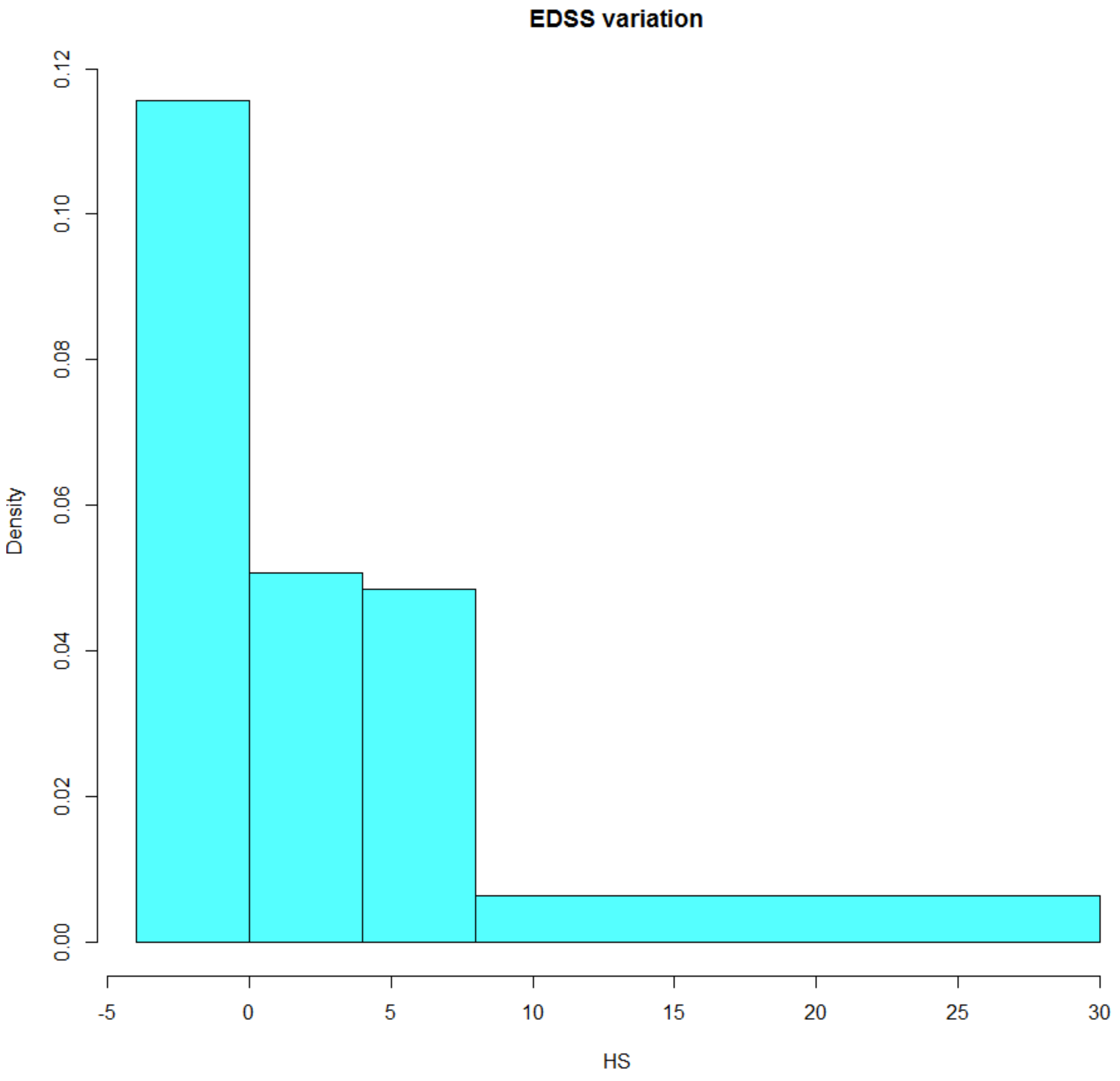

This histogram show the change in health status only due to EDSS variations between day 1 (baseline) and day 365 (last day of the study). The area of each bin is proportional to the number of patients. Roughly 20 % of patients had a clinically significant (more than ± 4.6 Health Status points) change of EDSS score with respect to their baseline.

SECTION 7. SENSITIVITY ANALYSIS USING BASELINE EDSS

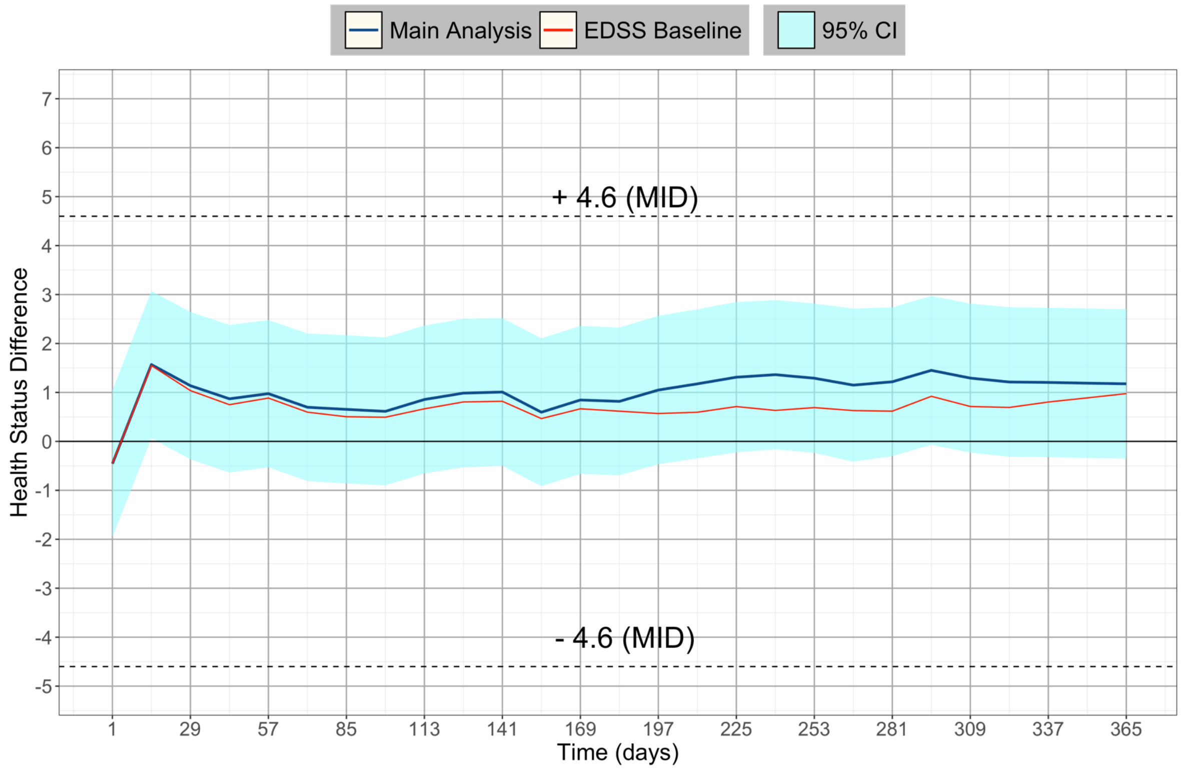

This sensitivity analysis shows the difference in health status between fingolimod 0.5mg and interferon beta-1a, when only the baseline values of EDSS are used neglecting EDSS follow up visits.

SECTION 8. ADVERSE EVENTS DROPS

Table 6 Collection of all AEs and related drops

	SOC_TXT[1]	*PT_TXT*[2]	*Drop*
1	Blood and lymphatic system disorders	Lymphadenopathy	7.5
2	Blood and lymphatic system disorders	Thrombocytopenia	7.5
3	Blood and lymphatic system disorders	Anaemia	7.5
4	Blood and lymphatic system disorders	Leukopenia	7.5
5	Blood and lymphatic system disorders	Lymphopenia	7.5
6	Blood and lymphatic system disorders	Macrocytosis	7.5
7	Blood and lymphatic system disorders	Thrombocythaemia	7.5
8	Blood and lymphatic system disorders	Haemorrhagic diathesis	7.5
9	Blood and lymphatic system disorders	Anaemia macrocytic	7.5
10	Blood and lymphatic system disorders	Eosinophilia	7.5
11	Blood and lymphatic system disorders	Monocytopenia	7.5
12	Blood and lymphatic system disorders	Polycythaemia	7.5
13	Blood and lymphatic system disorders	Lymphadenitis	7.5
14	Blood and lymphatic system disorders	Monocytosis	7.5
15	Blood and lymphatic system disorders	Neutropenia	7.5
16	Blood and lymphatic system disorders	Spontaneous haematoma	7.5
17	Blood and lymphatic system disorders	Haemorrhagic anaemia	7.5
18	Blood and lymphatic system disorders	Lymphadenopathy mediastinal	7.5
19	Blood and lymphatic system disorders	Haemoglobinaemia	7.5
20	Blood and lymphatic system disorders	Leukocytosis	7.5

21	Cardiac disorders	Atrioventricular block first degree	10
22	Cardiac disorders	Supraventricular extrasystoles	10
23	Cardiac disorders	Angina pectoris	10
24	Cardiac disorders	Palpitations	10
25	Cardiac disorders	Heart valve incompetence	10
26	Cardiac disorders	Bradycardia	10
27	Cardiac disorders	Myocardial infarction	10
28	Cardiac disorders	Ventricular tachycardia	10
29	Cardiac disorders	Bundle branch block right	10
30	Cardiac disorders	Atrioventricular block second degree	10
31	Cardiac disorders	Tachycardia	10
32	Cardiac disorders	Arrhythmia	10
33	Cardiac disorders	Cardiovascular disorder	10
34	Cardiac disorders	Left ventricular dysfunction	15
35	Cardiac disorders	Ventricular extrasystoles	15
36	Cardiac disorders	Pericarditis	15
37	Cardiac disorders	Diastolic dysfunction	15
38	Cardiac disorders	Coronary artery disease	15
39	Cardiac disorders	Tachycardia paroxysmal	15
40	Cardiac disorders	Bundle branch block left	15
41	Cardiac disorders	Extrasystoles	15
42	Cardiac disorders	Bradyarrhythmia	15
43	Cardiac disorders	Sinus bradycardia	15

44	Cardiac disorders	Arteriosclerosis coronary artery	15
45	Ear and labyrinth disorders	Tinnitus	10
46	Ear and labyrinth disorders	Vertigo positional	10
47	Ear and labyrinth disorders	Motion sickness	10
48	Ear and labyrinth disorders	Vertigo	10
49	Ear and labyrinth disorders	Ear pain	7.5
50	Ear and labyrinth disorders	Hypoacusis	7.5
51	Ear and labyrinth disorders	Ear discomfort	7.5
52	Ear and labyrinth disorders	Ear congestion	7.5
53	Ear and labyrinth disorders	Middle ear inflammation	10
54	Ear and labyrinth disorders	Tympanic membrane disorder	10
55	Ear and labyrinth disorders	Middle ear disorder	10
56	Ear and labyrinth disorders	Meniere's disease	10
57	Ear and labyrinth disorders	Tympanic membrane perforation	10
58	Ear and labyrinth disorders	Middle ear effusion	10
59	Ear and labyrinth disorders	Tympanic membrane hyperaemia	10
60	Ear and labyrinth disorders	Hyperacusis	7.5
61	Endocrine disorders	Goitre	10
62	Endocrine disorders	Hypothyroidism	10
63	Endocrine disorders	Hypogonadism	10
64	Endocrine disorders	Autoimmune thyroiditis	10
65	Endocrine disorders	Adrenal disorder	10
66	Eye disorders	Lacrimation increased	10

67	Eye disorders	Eye pain	7.5
68	Eye disorders	Visual acuity reduced	15
69	Eye disorders	Conjunctivitis	10
70	Eye disorders	Keratoconjunctivitis sicca	15
71	Eye disorders	Vision blurred	15
72	Eye disorders	Myopia	10
73	Eye disorders	Eye swelling	15
74	Eye disorders	Iritis	15
75	Eye disorders	Visual impairment	15
76	Eye disorders	Chorioretinopathy	15
77	Eye disorders	Retinal haemorrhage	15
78	Eye disorders	Conjunctivitis allergic	10
79	Eye disorders	Diplopia	15
80	Eye disorders	Scotoma	15
81	Eye disorders	Lacrimal disorder	10
82	Eye disorders	Conjunctival haemorrhage	7.5
83	Eye disorders	Asthenopia	7.5
84	Eye disorders	Dry eye	7.5
85	Eye disorders	Abnormal sensation in eye	7.5
86	Eye disorders	Blepharospasm	7.5
87	Eye disorders	Panophthalmitis	15
88	Eye disorders	Retinal disorder	15
89	Eye disorders	Photopsia	7.5
90	Eye disorders	Eye haemorrhage	15

91	Eye disorders	Dacryostenosis acquired	7.5
92	Eye disorders	Eyelid oedema	10
93	Eye disorders	Uhthoff's phenomenon	10
94	Eye disorders	Retinopathy	15
95	Eye disorders	Keratitis	15
96	Eye disorders	Iridocyclitis	10
97	Eye disorders	Eye disorder	15
98	Eye disorders	Optic atrophy	15
99	Eye disorders	Presbyopia	10
100	Eye disorders	Uveitis	15
101	Eye disorders	Cataract	7.5
102	Eye disorders	Blepharitis	7.5
103	Eye disorders	Macular oedema	20
104	Eye disorders	Ocular discomfort	7.5
105	Eye disorders	Corneal oedema	15
106	Eye disorders	Corneal disorder	15
107	Eye disorders	Eye irritation	7.5
108	Eye disorders	Ocular vascular disorder	15
109	Eye disorders	Posterior capsule opacification	15
110	Eye disorders	Papilloedema	15
111	Eye disorders	Strabismus	15
112	Eye disorders	Amblyopia	15
113	Eye disorders	Retinitis	15
114	Eye disorders	Myodesopsia	15

115	Eye disorders	Vitritis	10
116	Eye disorders	Lens disorder	15
117	Eye disorders	Retinal detachment	15
118	Eye disorders	Ophthalmoplegia	15
119	Eye disorders	Accommodation disorder	15
120	Eye disorders	Chalazion	7.5
121	Eye disorders	Scleral discolouration	7.5
122	Eye disorders	Eye pruritus	7.5
123	Eye disorders	Optic disc haemorrhage	15
124	Eye disorders	Maculopathy	15
125	Eye disorders	Retinal aneurysm	20
126	Eye disorders	Retinal pigmentation	7.5
127	Eye disorders	Macular degeneration	15
128	Eye disorders	Retinal pigment epitheliopathy	15
129	Eye disorders	Arteriosclerotic retinopathy	15
130	Eye disorders	Eyelid ptosis	10
131	Gastrointestinal disorders	Constipation	7.5
132	Gastrointestinal disorders	Gastrooesophageal reflux disease	7.5
133	Gastrointestinal disorders	Dyspepsia	7.5
134	Gastrointestinal disorders	Diarrhoea	7.5
135	Gastrointestinal disorders	Nausea	7.5
136	Gastrointestinal disorders	Vomiting	7.5
137	Gastrointestinal disorders	Toothache	7.5
138	Gastrointestinal disorders	Abdominal pain upper	7.5

139	Gastrointestinal disorders	Mouth ulceration	7.5
140	Gastrointestinal disorders	Faecal incontinence	20
141	Gastrointestinal disorders	Flatulence	7.5
142	Gastrointestinal disorders	Frequent bowel movements	7.5
143	Gastrointestinal disorders	Gingivitis	7.5
144	Gastrointestinal disorders	Paraesthesia oral	7.5
145	Gastrointestinal disorders	Abdominal distension	7.5
146	Gastrointestinal disorders	Periodontitis	7.5
147	Gastrointestinal disorders	Gastritis	10
148	Gastrointestinal disorders	Aphthous stomatitis	7.5
149	Gastrointestinal disorders	Pancreas lipomatosis	7.5
150	Gastrointestinal disorders	Dental caries	7.5
151	Gastrointestinal disorders	Enteritis	7.5
152	Gastrointestinal disorders	Haemorrhoids	7.5
153	Gastrointestinal disorders	Abdominal pain	7.5
154	Gastrointestinal disorders	Duodenogastric reflux	7.5
155	Gastrointestinal disorders	Peptic ulcer	7.5
156	Gastrointestinal disorders	Hypoaesthesia oral	7.5
157	Gastrointestinal disorders	Irritable bowel syndrome	15
158	Gastrointestinal disorders	Tooth disorder	7.5
159	Gastrointestinal disorders	Abdominal discomfort	7.5
160	Gastrointestinal disorders	Gingival bleeding	7.5
161	Gastrointestinal disorders	Food poisoning	7.5
162	Gastrointestinal disorders	Gastrointestinal motility disorder	7.5

163	Gastrointestinal disorders	Stomatitis	7.5
164	Gastrointestinal disorders	Tooth erosion	10
165	Gastrointestinal disorders	Eructation	7.5
166	Gastrointestinal disorders	Lip disorder	7.5
167	Gastrointestinal disorders	Epigastric discomfort	7.5
168	Gastrointestinal disorders	Dry mouth	7.5
169	Gastrointestinal disorders	Glossitis	7.5
170	Gastrointestinal disorders	Cheilitis	7.5
171	Gastrointestinal disorders	Bowel movement irregularity	7.5
172	Gastrointestinal disorders	Rectal haemorrhage	7.5
173	Gastrointestinal disorders	Anal pruritus	7.5
174	Gastrointestinal disorders	Tooth loss	10
175	Gastrointestinal disorders	Oral pain	7.5
176	Gastrointestinal disorders	Oesophageal pain	7.5
177	Gastrointestinal disorders	Intestinal polyp	7.5
178	Gastrointestinal disorders	Sensitivity of teeth	7.5
179	Gastrointestinal disorders	Rectal tenesmus	7.5
180	Gastrointestinal disorders	Colonic polyp	7.5
181	Gastrointestinal disorders	Intestinal functional disorder	7.5
182	Gastrointestinal disorders	Sigmoiditis	10
183	Gastrointestinal disorders	Abdominal pain lower	7.5
184	Gastrointestinal disorders	Glossodynia	7.5
185	Gastrointestinal disorders	Gastric polyps	7.5
186	Gastrointestinal disorders	Inguinal hernia	7.5

187	Gastrointestinal disorders	Reflux oesophagitis	7.5
188	Gastrointestinal disorders	Oesophagitis	7.5
189	Gastrointestinal disorders	Pancreatitis chronic	20
190	Gastrointestinal disorders	Haemorrhoidal haemorrhage	10
191	Gastrointestinal disorders	Tongue spasm	7.5
192	Gastrointestinal disorders	Tongue blistering	7.5
193	Gastrointestinal disorders	Tongue coated	7.5
194	Gastrointestinal disorders	Haematochezia	7.5
195	Gastrointestinal disorders	Lip swelling	7.5
196	Gastrointestinal disorders	Lip blister	7.5
197	Gastrointestinal disorders	Aptyalism	7.5
198	Gastrointestinal disorders	Salivary hypersecretion	7.5
199	Gastrointestinal disorders	Dysphagia	10
200	Gastrointestinal disorders	Salivary gland cyst	7.5
201	Gastrointestinal disorders	Coeliac disease	7.5
202	Gastrointestinal disorders	Breath odour	7.5
203	Gastrointestinal disorders	Oesophageal spasm	7.5
204	Gastrointestinal disorders	Diverticulum intestinal	10
205	Gastrointestinal disorders	Hiatus hernia	10
206	Gastrointestinal disorders	Proctalgia	7.5
207	Gastrointestinal disorders	Abdominal tenderness	7.5
208	Gastrointestinal disorders	Gastrointestinal disorder	7.5
209	Gastrointestinal disorders	Odynophagia	7.5
210	Gastrointestinal disorders	Defaecation urgency	15

211	Gastrointestinal disorders	Lip ulceration	7.5
212	Gastrointestinal disorders	Oral pruritus	7.5
213	Gastrointestinal disorders	Mouth cyst	7.5
214	Gastrointestinal disorders	Gingival pain	7.5
215	Gastrointestinal disorders	Palatal oedema	7.5
216	Gastrointestinal disorders	Ileus paralytic	20
217	General disorders and administration site conditions	No adverse event	0.1
218	General disorders and administration site conditions	Fatigue	10
219	General disorders and administration site conditions	Infusion site extravasation	7.5
220	General disorders and administration site conditions	Vessel puncture site pain	7.5
221	General disorders and administration site conditions	Chest discomfort	7.5
222	General disorders and administration site conditions	Pain	7.5
223	General disorders and administration site conditions	Gait disturbance	15
224	General disorders and administration site conditions	Non-cardiac chest pain	7.5
225	General disorders and administration site conditions	Sensation of foreign body	7.5
226	General disorders and administration site conditions	Cyst	7.5
227	General disorders and administration site conditions	Malaise	7.5
228	General disorders and administration site conditions	Pyrexia	10
229	General disorders and administration site conditions	Facial pain	7.5
230	General disorders and administration site conditions	Oedema peripheral	7.5

231	General disorders and administration site conditions	Asthenia	7.5
232	General disorders and administration site conditions	Influenza like illness	10
233	General disorders and administration site conditions	Feeling cold	7.5
234	General disorders and administration site conditions	Oedema mucosal	7.5
235	General disorders and administration site conditions	General physical health deterioration	20
236	General disorders and administration site conditions	Calcinosis	7.5
237	General disorders and administration site conditions	Injection site irritation	7.5
238	General disorders and administration site conditions	Haemorrhagic cyst	7.5
239	General disorders and administration site conditions	Irritability	7.5
240	General disorders and administration site conditions	Infusion related reaction	7.5
241	General disorders and administration site conditions	Local swelling	7.5
242	General disorders and administration site conditions	Injection site swelling	7.5
243	General disorders and administration site conditions	Secretion discharge	7.5
244	General disorders and administration site conditions	Xerosis	7.5
245	General disorders and administration site conditions	Chest pain	7.5
246	General disorders and administration site conditions	Hyperthermia	7.5
247	General disorders and administration site conditions	Injection site rash	7.5
248	General disorders and administration site conditions	Impaired healing	7.5

249	General disorders and administration site conditions	Exercise tolerance decreased	7.5
250	General disorders and administration site conditions	Feeling hot	7.5
251	General disorders and administration site conditions	Hangover	7.5
252	General disorders and administration site conditions	Feeling of body temperature change	7.5
253	General disorders and administration site conditions	Chills	7.5
254	General disorders and administration site conditions	Injection site haematoma	7.5
255	General disorders and administration site conditions	Face oedema	7.5
256	General disorders and administration site conditions	Obstruction	7.5
257	General disorders and administration site conditions	Oedema	7.5
258	General disorders and administration site conditions	Axillary pain	7.5
259	General disorders and administration site conditions	Discomfort	7.5
260	General disorders and administration site conditions	Inflammation	7.5
261	General disorders and administration site conditions	Injection site reaction	7.5
262	General disorders and administration site conditions	Injection site pain	7.5
263	General disorders and administration site conditions	Mucosal exfoliation	7.5
264	General disorders and administration site conditions	Hunger	7.5
265	General disorders and administration site conditions	Thirst	7.5
266	General disorders and administration site conditions	Temperature intolerance	7.5

267	General disorders and administration site conditions	Infusion site irritation	7.5
268	General disorders and administration site conditions	Meteoropathy	7.5
269	General disorders and administration site conditions	Nodule	7.5
270	General disorders and administration site conditions	Fat tissue increased	10
271	General disorders and administration site conditions	Generalised oedema	10
272	Hepatobiliary disorders	Biliary colic	10
273	Hepatobiliary disorders	Cholecystitis	10
274	Hepatobiliary disorders	Hyperbilirubinaemia	7.5
275	Hepatobiliary disorders	Liver disorder	10
276	Hepatobiliary disorders	Cholelithiasis	10
277	Hepatobiliary disorders	Hepatic steatosis	10
278	Hepatobiliary disorders	Cholecystitis chronic	10
279	Hepatobiliary disorders	Gallbladder disorder	7.5
280	Hepatobiliary disorders	Hepatomegaly	10
281	Hepatobiliary disorders	Cytolytic hepatitis	10
282	Hepatobiliary disorders	Gallbladder polyp	7.5
283	Hepatobiliary disorders	Bile duct stone	10
284	Hepatobiliary disorders	Jaundice cholestatic	10
285	Hepatobiliary disorders	Hepatic function abnormal	7.5
286	Hepatobiliary disorders	Hepatic pain	10
287	Immune system disorders	Seasonal allergy	7.5
288	Immune system disorders	Drug hypersensitivity	10

289	Immune system disorders	Allergy to animal	7.5
290	Immune system disorders	Food allergy	7.5
291	Immune system disorders	Multiple allergies	10
292	Immune system disorders	Allergy to arthropod bite	7.5
293	Immune system disorders	Hypersensitivity	10
294	Immune system disorders	Atopy	10
295	Infections and infestations	Lower respiratory tract infection	10
296	Infections and infestations	Upper respiratory tract infection	10
297	Infections and infestations	Bronchitis	10
298	Infections and infestations	Fungal infection	10
299	Infections and infestations	Viral upper respiratory tract infection	10
300	Infections and infestations	Tooth abscess	10
301	Infections and infestations	Pharyngitis	10
302	Infections and infestations	Cellulitis	10
303	Infections and infestations	Impetigo	10
304	Infections and infestations	Gastroenteritis	10
305	Infections and infestations	Sinusitis	10
306	Infections and infestations	Nasopharyngitis	10
307	Infections and infestations	Nail infection	10
308	Infections and infestations	Influenza	10
309	Infections and infestations	Gingival infection	10
310	Infections and infestations	Pertussis	20
311	Infections and infestations	Urinary tract infection	10

312	Infections and infestations	Papilloma viral infection	10
313	Infections and infestations	Viral infection	15
314	Infections and infestations	Furuncle	10
315	Infections and infestations	Herpes virus infection	10
316	Infections and infestations	Laryngitis	10
317	Infections and infestations	Perineal abscess	10
318	Infections and infestations	Respiratory tract infection	10
319	Infections and infestations	Lower respiratory tract infection viral	10
320	Infections and infestations	Cystitis	7.5
321	Infections and infestations	Vaginal infection	10
322	Infections and infestations	Mastitis	10
323	Infections and infestations	Folliculitis	7.5
324	Infections and infestations	Pulpitis dental	7.5
325	Infections and infestations	Rhinitis	7.5
326	Infections and infestations	Tonsillitis	7.5
327	Infections and infestations	Herpes zoster	10
328	Infections and infestations	Vulvovaginal mycotic infection	10
329	Infections and infestations	Oral herpes	7.5
330	Infections and infestations	Genital herpes	10
331	Infections and infestations	Pneumonia	10
332	Infections and infestations	Campylobacter gastroenteritis	10
333	Infections and infestations	Adenovirus infection	10
334	Infections and infestations	Otitis media	7.5

335	Infections and infestations	Herpes simplex	7.5
336	Infections and infestations	Acute tonsillitis	7.5
337	Infections and infestations	Tinea versicolour	7.5
338	Infections and infestations	Tracheitis	7.5
339	Infections and infestations	Urosepsis	10
340	Infections and infestations	Bronchiolitis	10
341	Infections and infestations	Tinea pedis	7.5
342	Infections and infestations	Onychomycosis	7.5
343	Infections and infestations	Tooth infection	7.5
344	Infections and infestations	Bronchopneumonia	10
345	Infections and infestations	Gastrointestinal infection	10
346	Infections and infestations	Rhinolaryngitis	7.5
347	Infections and infestations	Vulvovaginitis	7.5
348	Infections and infestations	Respiratory tract infection viral	10
349	Infections and infestations	Anogenital warts	7.5
350	Infections and infestations	Rash pustular	10
351	Infections and infestations	Osteomyelitis	15
352	Infections and infestations	Gastritis viral	15
353	Infections and infestations	Gastroenteritis viral	15
354	Infections and infestations	Groin abscess	10
355	Infections and infestations	Borrelia infection	10
356	Infections and infestations	Enterobiasis	10
357	Infections and infestations	Helminthic infection	10
358	Infections and infestations	Peritonsillitis	10

359	Infections and infestations	Gastrointestinal fungal infection	15
360	Infections and infestations	Abscess	10
361	Infections and infestations	Fungal skin infection	10
362	Infections and infestations	Oral candidiasis	10
363	Infections and infestations	Breast abscess	10
364	Infections and infestations	Mucosal infection	10
365	Infections and infestations	Ear infection	10
366	Infections and infestations	Lung infection	20
367	Infections and infestations	Acute haemorrhagic conjunctivitis	10
368	Infections and infestations	Alveolar osteitis	10
369	Infections and infestations	Sinobronchitis	10
370	Infections and infestations	Paronychia	10
371	Infections and infestations	Genital infection	10
372	Infections and infestations	Dermo-hypodermitis	10
373	Infections and infestations	Streptococcal abscess	10
374	Infections and infestations	Pyelonephritis	10
375	Infections and infestations	Candidiasis	10
376	Infections and infestations	Laryngitis viral	10
377	Infections and infestations	Anal abscess	10
378	Infections and infestations	Bronchitis viral	15
379	Infections and infestations	Vulvovaginal candidiasis	10
380	Infections and infestations	Vulvovaginitis trichomonal	10
381	Infections and infestations	Genitourinary tract infection	15

382	Infections and infestations	Lice infestation	10
383	Infections and infestations	Otitis externa	10
384	Infections and infestations	Conjunctivitis viral	10
385	Infections and infestations	Peritonsillar abscess	10
386	Infections and infestations	Viral rhinitis	10
387	Infections and infestations	Erysipelas	10
388	Infections and infestations	Abscess jaw	10
389	Infections and infestations	Subcutaneous abscess	10
390	Infections and infestations	Epidemic pleurodynia	10
391	Infections and infestations	Localised infection	10
392	Infections and infestations	Eye infection	10
393	Infections and infestations	Lyme disease	10
394	Infections and infestations	Eye infection fungal	10
395	Infections and infestations	Vestibular neuronitis	10
396	Infections and infestations	Chronic sinusitis	10
397	Infections and infestations	Eczema infected	10
398	Infections and infestations	Helicobacter infection	10
399	Infections and infestations	Oral fungal infection	10
400	Infections and infestations	Erythema migrans	10
401	Infections and infestations	Salpingo-oophoritis	10
402	Infections and infestations	Clostridial infection	10
403	Infections and infestations	Staphylococcal impetigo	10
404	Infections and infestations	Molluscum contagiosum	10
405	Infections and infestations	Appendicitis	15

406	Infections and infestations	Peritoneal abscess	15
407	Infections and infestations	Diverticulitis	15
408	Infections and infestations	Pyelonephritis acute	15
409	Infections and infestations	Otitis media acute	10
410	Infections and infestations	Acute sinusitis	10
411	Infections and infestations	Mastoiditis	10
412	Infections and infestations	Pyelonephritis chronic	15
413	Infections and infestations	Hordeolum	10
414	Infections and infestations	Vaginitis bacterial	7.5
415	Infections and infestations	Skin infection	10
416	Infections and infestations	Salpingitis	15
417	Infections and infestations	Pneumonia mycoplasmal	10
418	Infections and infestations	Acarodermatitis	10
419	Infections and infestations	Pharyngotonsillitis	10
420	Infections and infestations	Pneumonia primary atypical	15
421	Infections and infestations	Infection	10
422	Infections and infestations	Orchitis	10
423	Infections and infestations	Tinea infection	10
424	Infections and infestations	Viraemia	20
425	Infections and infestations	Conjunctivitis infective	10
426	Infections and infestations	Pharyngitis streptococcal	10
427	Infections and infestations	Infected epidermal cyst	10
428	Infections and infestations	Genital infection female	10
429	Infections and infestations	Bacteriuria	10

430	Infections and infestations	Herpes dermatitis	10
431	Infections and infestations	Body tinea	10
432	Infections and infestations	Anal infection	10
433	Infections and infestations	West Nile viral infection	20
434	Infections and infestations	Kidney infection	20
435	Infections and infestations	Oral viral infection	10
436	Infections and infestations	Enterocolitis infectious	10
437	Injury, poisoning and procedural complications	Ligament injury	15
443	Injury, poisoning and procedural complications	Limb injury	15
444	Injury, poisoning and procedural complications	Excoriation	7.5
445	Injury, poisoning and procedural complications	Joint sprain	15
446	Injury, poisoning and procedural complications	Traumatic shock	15
447	Injury, poisoning and procedural complications	Wound	15
448	Injury, poisoning and procedural complications	Epicondylitis	10
449	Injury, poisoning and procedural complications	Procedural pain	15
450	Injury, poisoning and procedural complications	Meniscus lesion	20
451	Injury, poisoning and procedural complications	Subdural haematoma	20
452	Injury, poisoning and procedural complications	Humerus fracture	15
453	Injury, poisoning and procedural complications	Joint injury	15
454	Injury, poisoning and procedural complications	Head injury	15

455	Injury, poisoning and procedural complications	Tooth fracture	15
456	Injury, poisoning and procedural complications	Concussion	15
457	Injury, poisoning and procedural complications	Ligament rupture	15
458	Injury, poisoning and procedural complications	Injury	10
459	Injury, poisoning and procedural complications	Injury corneal	15
460	Injury, poisoning and procedural complications	Whiplash injury	15
461	Injury, poisoning and procedural complications	Tendon rupture	15
462	Injury, poisoning and procedural complications	Lower limb fracture	15
463	Injury, poisoning and procedural complications	Foot fracture	15
464	Injury, poisoning and procedural complications	Road traffic accident	15
465	Injury, poisoning and procedural complications	Muscle rupture	15
466	Injury, poisoning and procedural complications	Skeletal injury	15
467	Injury, poisoning and procedural complications	Ligament sprain	7.5
468	Injury, poisoning and procedural complications	Subcutaneous haematoma	7.5
469	Injury, poisoning and procedural complications	Hand fracture	15
470	Injury, poisoning and procedural complications	Eye luxation	15
471	Injury, poisoning and procedural complications	Ear injury	15
472	Injury, poisoning and procedural complications	Accident	15

473	Injury, poisoning and procedural complications	Fractured coccyx	15
474	Injury, poisoning and procedural complications	Post concussion syndrome	15
475	Injury, poisoning and procedural complications	Chillblains	7.5
476	Injury, poisoning and procedural complications	Upper limb fracture	15
477	Injury, poisoning and procedural complications	Scratch	7.5
478	Injury, poisoning and procedural complications	Foreign body trauma	15
479	Injury, poisoning and procedural complications	Bite	7.5
480	Injury, poisoning and procedural complications	Facial bones fracture	15
481	Injury, poisoning and procedural complications	Traumatic haematoma	15
482	Injury, poisoning and procedural complications	Open wound	15
483	Injury, poisoning and procedural complications	Ulnar nerve injury	15
484	Injury, poisoning and procedural complications	Back injury	15
485	Injury, poisoning and procedural complications	Splenic injury	15
486	Injury, poisoning and procedural complications	Overdose	15
487	Injury, poisoning and procedural complications	Forearm fracture	15
488	Injury, poisoning and procedural complications	Sunburn	7.5
489	Injury, poisoning and procedural complications	Post-traumatic pain	7.5
490	Injury, poisoning and procedural complications	Ankle fracture	15

491	Injury, poisoning and procedural complications	Rib fracture	15
492	Injury, poisoning and procedural complications	Burns second degree	15
493	Injury, poisoning and procedural complications	Splenic rupture	15
494	Injury, poisoning and procedural complications	Ulna fracture	15
495	Injury, poisoning and procedural complications	Tendon injury	10
496	Injury, poisoning and procedural complications	Muscle injury	10
497	Injury, poisoning and procedural complications	Post lumbar puncture syndrome	15
498	Injury, poisoning and procedural complications	Periorbital haematoma	15
499	Injury, poisoning and procedural complications	Clavicle fracture	15
500	Injury, poisoning and procedural complications	Muscle strain	7.5
501	Injury, poisoning and procedural complications	Corneal abrasion	15
502	Injury, poisoning and procedural complications	Medication error	7.5
503	Investigations	Liver function test abnormal	10
504	Investigations	Weight increased	10
505	Investigations	Electrocardiogram T wave amplitude decreased	10
506	Investigations	Electrocardiogram T wave inversion	10
507	Investigations	Weight decreased	10
508	Investigations	Hepatic enzyme increased	10
509	Investigations	Alanine aminotransferase increased	10

510	Investigations	Aspartate aminotransferase increased	10
511	Investigations	Gamma-glutamyltransferase increased	10
512	Investigations	White blood cell count decreased	10
513	Investigations	Biopsy prostate	10
514	Investigations	Blood folate decreased	10
515	Investigations	Borrelia burgdorferi serology positive	10
516	Investigations	Pulmonary function test decreased	10
517	Investigations	Laboratory test abnormal	10
518	Investigations	Gamma-glutamyltransferase abnormal	10
519	Investigations	Blood alkaline phosphatase abnormal	10
520	Investigations	Haemoglobin urine	10
521	Investigations	Blood amylase increased	10
522	Investigations	Blood cholesterol increased	10
523	Investigations	High density lipoprotein increased	10
524	Investigations	Transaminases increased	10
525	Investigations	Low density lipoprotein increased	10
526	Investigations	Blood triglycerides increased	10
527	Investigations	Lymphocyte count decreased	10
528	Investigations	Neutrophil count decreased	10
529	Investigations	Haemoglobin decreased	10
530	Investigations	Red blood cell count decreased	10

531	Investigations	Monocyte count increased	10
532	Investigations	Blood alkaline phosphatase increased	10
533	Investigations	Haematocrit decreased	10
534	Investigations	Monocyte count decreased	10
535	Investigations	Eosinophil count increased	10
536	Investigations	Precancerous cells present	10
537	Investigations	Low density lipoprotein decreased	10
538	Investigations	Carbon monoxide diffusing capacity decreased	10
539	Investigations	Neurological examination abnormal	10
540	Investigations	Blood urine present	10
541	Investigations	Platelet count decreased	10
542	Investigations	Heart rate increased	10
543	Investigations	Blood bilirubin increased	10
544	Investigations	Red blood cell sedimentation rate increased	10
545	Investigations	Blood pressure increased	10
546	Investigations	Mammogram	10
547	Investigations	Urine leukocyte esterase positive	10
548	Investigations	Computerised tomogram abnormal	10
549	Investigations	Spirometry	10
550	Investigations	Blood glucose increased	10
551	Investigations	Forced expiratory volume decreased	10
552	Investigations	Vital capacity decreased	10

553	Investigations	Blood pressure decreased	10
554	Investigations	Heart rate decreased	10
555	Investigations	Blood pressure systolic decreased	10
556	Investigations	Alanine aminotransferase decreased	10
557	Investigations	Blood pressure diastolic decreased	10
558	Investigations	Computerised tomogram thorax abnormal	10
559	Investigations	Activated partial thromboplastin time prolonged	10
560	Investigations	Total lung capacity increased	10
561	Investigations	Smear vaginal abnormal	10
562	Investigations	Electrocardiogram change	10
563	Investigations	Body mass index increased	10
564	Investigations	Electrocardiogram T wave abnormal	15
565	Investigations	Colonoscopy	10
566	Investigations	Pulmonary function test abnormal	10
567	Investigations	X-ray abnormal	10
568	Investigations	Lipoprotein (a) increased	10
569	Investigations	Blood pressure diastolic increased	10
570	Investigations	Macular reflex abnormal	10
571	Investigations	Blood carbon monoxide increased	10
572	Investigations	Bilirubin conjugated increased	10
573	Investigations	Smear cervix abnormal	10

574	Investigations	Electrocardiogram PQ interval	10
575	Investigations	Platelet count increased	10
576	Investigations	Blood alkaline phosphatase	10
577	Investigations	Intraocular pressure increased	10
578	Investigations	Electrocardiogram PR prolongation	10
579	Investigations	Heart rate irregular	10
580	Investigations	Mean cell volume abnormal	10
581	Investigations	Mean cell volume increased	10
582	Investigations	Blood creatinine increased	10
583	Investigations	Neutrophil count increased	10
584	Investigations	Nuclear magnetic resonance imaging abnormal	10
585	Investigations	Heart rate abnormal	10
586	Metabolism and nutrition disorders	Decreased appetite	7.5
587	Metabolism and nutrition disorders	Hypercholesterolaemia	7.5
588	Metabolism and nutrition disorders	Hyperlipidaemia	7.5
589	Metabolism and nutrition disorders	Dehydration	7.5
590	Metabolism and nutrition disorders	Hyperglycaemia	7.5
591	Metabolism and nutrition disorders	Hypokalaemia	7.5
592	Metabolism and nutrition disorders	Vitamin B12 deficiency	7.5
593	Metabolism and nutrition disorders	Iron deficiency	7.5
594	Metabolism and nutrition disorders	Hypertriglyceridaemia	7.5
595	Metabolism and nutrition disorders	Dyslipidaemia	7.5
596	Metabolism and nutrition disorders	Anorexia	7.5

597	Metabolism and nutrition disorders	Increased appetite	7.5
598	Metabolism and nutrition disorders	Diabetes mellitus	7.5
599	Metabolism and nutrition disorders	Hypoglycaemia	7.5
600	Metabolism and nutrition disorders	Hypovitaminosis	7.5
601	Metabolism and nutrition disorders	Malnutrition	10
602	Metabolism and nutrition disorders	Glucose tolerance impaired	15
603	Metabolism and nutrition disorders	Polydipsia	7.5
604	Metabolism and nutrition disorders	Gout	15
605	Metabolism and nutrition disorders	Calcium deficiency	7.5
606	Metabolism and nutrition disorders	Fluid retention	7.5
607	Musculoskeletal and connective tissue disorders	Back pain	10
608	Musculoskeletal and connective tissue disorders	Muscle spasms	10
609	Musculoskeletal and connective tissue disorders	Joint swelling	10
610	Musculoskeletal and connective tissue disorders	Musculoskeletal stiffness	10
611	Musculoskeletal and connective tissue disorders	Pain in jaw	10
612	Musculoskeletal and connective tissue disorders	Neck pain	10
613	Musculoskeletal and connective tissue disorders	Musculoskeletal discomfort	10
614	Musculoskeletal and connective tissue disorders	Arthralgia	10
615	Musculoskeletal and connective tissue disorders	Arthritis	10
616	Musculoskeletal and connective tissue disorders	Musculoskeletal chest pain	10
617	Musculoskeletal and connective tissue disorders	Groin pain	10

618	Musculoskeletal and connective tissue disorders	Pain in extremity	10
619	Musculoskeletal and connective tissue disorders	Tendonitis	10
620	Musculoskeletal and connective tissue disorders	Muscle twitching	10
621	Musculoskeletal and connective tissue disorders	Sympathetic posterior cervical syndrome	10
622	Musculoskeletal and connective tissue disorders	Osteoarthritis	10
623	Musculoskeletal and connective tissue disorders	Synovitis	10
624	Musculoskeletal and connective tissue disorders	Tenosynovitis	10
625	Musculoskeletal and connective tissue disorders	Patellofemoral pain syndrome	10
626	Musculoskeletal and connective tissue disorders	Arthropathy	10
627	Musculoskeletal and connective tissue disorders	Osteoporosis	10
628	Musculoskeletal and connective tissue disorders	Bone pain	10
629	Musculoskeletal and connective tissue disorders	Exostosis	10
630	Musculoskeletal and connective tissue disorders	Musculoskeletal pain	10
631	Musculoskeletal and connective tissue disorders	Intervertebral disc protrusion	10
632	Musculoskeletal and connective tissue disorders	Muscular weakness	10
633	Musculoskeletal and connective tissue disorders	Myalgia	10
634	Musculoskeletal and connective tissue disorders	Bursitis	10
635	Musculoskeletal and connective tissue disorders	Tendon pain	10

636	Musculoskeletal and connective tissue disorders	Ligament disorder	10
637	Musculoskeletal and connective tissue disorders	Trigger finger	10
638	Musculoskeletal and connective tissue disorders	Foot deformity	10
639	Musculoskeletal and connective tissue disorders	Joint stiffness	10
640	Musculoskeletal and connective tissue disorders	Myositis	10
641	Musculoskeletal and connective tissue disorders	Sensation of heaviness	10
642	Musculoskeletal and connective tissue disorders	Torticollis	10
643	Musculoskeletal and connective tissue disorders	Muscle tightness	10
644	Musculoskeletal and connective tissue disorders	Jaw cyst	10
645	Musculoskeletal and connective tissue disorders	Muscle contracture	10
646	Musculoskeletal and connective tissue disorders	Tendon disorder	10
647	Musculoskeletal and connective tissue disorders	Joint effusion	10
648	Musculoskeletal and connective tissue disorders	Rotator cuff syndrome	10
649	Musculoskeletal and connective tissue disorders	Synovial cyst	10
650	Musculoskeletal and connective tissue disorders	Flank pain	10
651	Musculoskeletal and connective tissue disorders	Mobility decreased	15
652	Musculoskeletal and connective tissue disorders	Muscle atrophy	10
653	Musculoskeletal and connective tissue disorders	Spinal osteoarthritis	10

654	Musculoskeletal and connective tissue disorders	Metatarsalgia	10
655	Musculoskeletal and connective tissue disorders	Intervertebral disc disorder	10
656	Musculoskeletal and connective tissue disorders	Bone cyst	10
657	Musculoskeletal and connective tissue disorders	Osteitis	10
658	Musculoskeletal and connective tissue disorders	Myokymia	10
659	Musculoskeletal and connective tissue disorders	Costochondritis	10
660	Musculoskeletal and connective tissue disorders	Joint lock	10
661	Musculoskeletal and connective tissue disorders	Limb discomfort	10
662	Musculoskeletal and connective tissue disorders	Morphoea	10
663	Musculoskeletal and connective tissue disorders	Nodule on extremity	10
664	Musculoskeletal and connective tissue disorders	Plantar fasciitis	10
665	Musculoskeletal and connective tissue disorders	Intervertebral disc degeneration	10
666	Musculoskeletal and connective tissue disorders	Chondritis	10
667	Musculoskeletal and connective tissue disorders	Medial tibial stress syndrome	10
668	Musculoskeletal and connective tissue disorders	Rheumatoid arthritis	15
669	Musculoskeletal and connective tissue disorders	Coccydynia	10
670	Neoplasms benign, malignant and unspecified (incl cysts and polyps)	Basal cell carcinoma	10
671	Neoplasms benign, malignant and unspecified (incl cysts and polyps)	Benign neoplasm of skin	10

672	Neoplasms benign, malignant and unspecified (incl cysts and polyps)	Bowen's disease	10
673	Neoplasms benign, malignant and unspecified (incl cysts and polyps)	Seborrhoeic keratosis	10
674	Neoplasms benign, malignant and unspecified (incl cysts and polyps)	Skin papilloma	10
675	Neoplasms benign, malignant and unspecified (incl cysts and polyps)	Malignant melanoma	20
676	Neoplasms benign, malignant and unspecified (incl cysts and polyps)	Melanocytic naevus	10
677	Neoplasms benign, malignant and unspecified (incl cysts and polyps)	Prostate cancer	20
678	Neoplasms benign, malignant and unspecified (incl cysts and polyps)	Uterine leiomyoma	10
679	Neoplasms benign, malignant and unspecified (incl cysts and polyps)	Benign lymph node neoplasm	10
680	Neoplasms benign, malignant and unspecified (incl cysts and polyps)	Fibroadenoma of breast	10
681	Neoplasms benign, malignant and unspecified (incl cysts and polyps)	Lipoma	10
682	Neoplasms benign, malignant and unspecified (incl cysts and polyps)	Benign neoplasm	10
683	Neoplasms benign, malignant and unspecified (incl cysts and polyps)	Brain neoplasm benign	10
684	Neoplasms benign, malignant and unspecified (incl cysts and polyps)	Benign ovarian tumour	10
685	Neoplasms benign, malignant and unspecified (incl cysts and polyps)	Lipofibroma	10
686	Neoplasms benign, malignant and unspecified (incl cysts and polyps)	Benign breast neoplasm	10
687	Neoplasms benign, malignant and unspecified (incl cysts and polyps)	Becker's naevus	10
688	Neoplasms benign, malignant and unspecified (incl cysts and polyps)	Breast cancer	10
689	Neoplasms benign, malignant and unspecified (incl cysts and polyps)	Meningioma benign	10

690	Neoplasms benign, malignant and unspecified (incl cysts and polyps)	Benign hepatic neoplasm	10
691	Neoplasms benign, malignant and unspecified (incl cysts and polyps)	Hair follicle tumour benign	10
692	Neoplasms benign, malignant and unspecified (incl cysts and polyps)	Benign muscle neoplasm	10
693	Neoplasms benign, malignant and unspecified (incl cysts and polyps)	Haemangioma	10
694	Neoplasms benign, malignant and unspecified (incl cysts and polyps)	Colon adenoma	10
695	Neoplasms benign, malignant and unspecified (incl cysts and polyps)	Adenoma benign	10
696	Neoplasms benign, malignant and unspecified (incl cysts and polyps)	Benign lung neoplasm	10
697	Neoplasms benign, malignant and unspecified (incl cysts and polyps)	Angiomyolipoma	10
698	Neoplasms benign, malignant and unspecified (incl cysts and polyps)	Lipoma of breast	10
699	Neoplasms benign, malignant and unspecified (incl cysts and polyps)	Oral papilloma	10
700	Neoplasms benign, malignant and unspecified (incl cysts and polyps)	Ovarian adenoma	10
701	Neoplasms benign, malignant and unspecified (incl cysts and polyps)	Benign renal neoplasm	10
702	Neoplasms benign, malignant and unspecified (incl cysts and polyps)	Cervix carcinoma stage 0	10
703	Neoplasms benign, malignant and unspecified (incl cysts and polyps)	Fibroma	10
704	Neoplasms benign, malignant and unspecified (incl cysts and polyps)	Eye naevus	10
705	Neoplasms benign, malignant and unspecified (incl cysts and polyps)	Fibrous histiocytoma	10
706	Neoplasms benign, malignant and unspecified (incl cysts and polyps)	Endometrial cancer	20
707	Neoplasms benign, malignant and unspecified (incl cysts and polyps)	Paranasal sinus benign neoplasm	10

708	Neoplasms benign, malignant and unspecified (incl cysts and polyps)	Benign anorectal neoplasm	10
709	Neoplasms benign, malignant and unspecified (incl cysts and polyps)	Haemangioma of skin	10
710	Neoplasms benign, malignant and unspecified (incl cysts and polyps)	Acrochordon	10
711	Neoplasms benign, malignant and unspecified (incl cysts and polyps)	Benign pleural neoplasm	10
712	Neoplasms benign, malignant and unspecified (incl cysts and polyps)	Lung neoplasm	20
713	Nervous system disorders	Headache	7.5
714	Nervous system disorders	Optic neuritis	20
715	Nervous system disorders	Dizziness	7.5
716	Nervous system disorders	Hypoaesthesia	10
717	Nervous system disorders	Migraine	7.5
718	Nervous system disorders	Memory impairment	20
719	Nervous system disorders	Carpal tunnel syndrome	10
720	Nervous system disorders	Migraine with aura	10
721	Nervous system disorders	Balance disorder	20
722	Nervous system disorders	Trigeminal neuralgia	10
723	Nervous system disorders	Paraesthesia	10
724	Nervous system disorders	Hyperaesthesia	10
725	Nervous system disorders	Cognitive disorder	15
726	Nervous system disorders	Ataxia	10
727	Nervous system disorders	Burning sensation	10
728	Nervous system disorders	Sciatica	10
729	Nervous system disorders	Epilepsy	20

730	Nervous system disorders	Tremor	10
731	Nervous system disorders	Neuralgia	10
732	Nervous system disorders	Cluster headache	10
733	Nervous system disorders	Lumbar radiculopathy	10
734	Nervous system disorders	Radiculopathy	10
735	Nervous system disorders	Cervicobrachial syndrome	10
736	Nervous system disorders	Autonomic nervous system imbalance	10
737	Nervous system disorders	Presyncope	10
738	Nervous system disorders	Syncope	10
739	Nervous system disorders	Facial neuralgia	10
740	Nervous system disorders	Dysaesthesia	10
741	Nervous system disorders	Intracranial haematoma	20
742	Nervous system disorders	Brain oedema	20
743	Nervous system disorders	Tension headache	10
744	Nervous system disorders	Restless legs syndrome	10
745	Nervous system disorders	Motor dysfunction	15
746	Nervous system disorders	Coordination abnormal	15
747	Nervous system disorders	Peroneal nerve palsy	15
748	Nervous system disorders	Dysphasia	15
749	Nervous system disorders	Dysgeusia	15
750	Nervous system disorders	Sensory disturbance	15
751	Nervous system disorders	Hypotonia	10
752	Nervous system disorders	Brain stem syndrome	15
753	Nervous system disorders	Aphasia	15

754	Nervous system disorders	Disturbance in attention	15
755	Nervous system disorders	Amnesia	20
756	Nervous system disorders	Hypertonia	10
757	Nervous system disorders	Lethargy	10
758	Nervous system disorders	Muscle spasticity	10
759	Nervous system disorders	Loss of consciousness	15
760	Nervous system disorders	Somnolence	10
761	Nervous system disorders	Partial seizures	20
762	Nervous system disorders	Partial seizures with secondary generalisation	20
763	Nervous system disorders	Subarachnoid haemorrhage	20
764	Nervous system disorders	Cervical root pain	7.5
765	Nervous system disorders	Speech disorder	15
766	Nervous system disorders	Cranial neuropathy	20
767	Nervous system disorders	Lhermitte's sign	7.5
768	Nervous system disorders	Hypersomnia	10
769	Nervous system disorders	Radicular pain	10
770	Nervous system disorders	Hemianopia homonymous	20
771	Nervous system disorders	Neurological symptom	10
772	Nervous system disorders	Hemiparesis	20
773	Nervous system disorders	Monoparesis	20
774	Nervous system disorders	Radicular syndrome	10
775	Nervous system disorders	Muscle contractions involuntary	7.5
776	Nervous system disorders	Hyperpathia	15

777	Nervous system disorders	Dyskinesia	10
778	Nervous system disorders	Amnestic disorder	20
779	Nervous system disorders	Grand mal convulsion	20
780	Nervous system disorders	Facial palsy	10
781	Nervous system disorders	Nerve compression	10
782	Nervous system disorders	Radiculitis lumbosacral	10
783	Nervous system disorders	Neuropathy peripheral	10
784	Nervous system disorders	Monoplegia	20
785	Nervous system disorders	Dizziness postural	7.5
786	Nervous system disorders	Petit mal epilepsy	20
787	Nervous system disorders	Cerebrovascular accident	20
788	Nervous system disorders	Hemianopia	20
789	Nervous system disorders	Parosmia	7.5
790	Nervous system disorders	Central nervous system lesion	20
791	Nervous system disorders	Ischaemic stroke	20
792	Nervous system disorders	Clumsiness	7.5
793	Nervous system disorders	Head discomfort	7.5
794	Nervous system disorders	Dysarthria	15
795	Nervous system disorders	Sinus headache	7.5
796	Nervous system disorders	Sensory loss	20
797	Nervous system disorders	Perineurial cyst	10
798	Pregnancy, puerperium and perinatal conditions	Abortion	15
799	Pregnancy, puerperium and perinatal conditions	Pregnancy	10

800	Pregnancy, puerperium and perinatal conditions	Abortion spontaneous	15
801	Psychiatric disorders	Mood altered	10
802	Psychiatric disorders	Insomnia	15
803	Psychiatric disorders	Depression	15
804	Psychiatric disorders	Libido decreased	7.5
805	Psychiatric disorders	Homicidal ideation	20
806	Psychiatric disorders	Bipolar disorder	20
807	Psychiatric disorders	Anxiety	15
808	Psychiatric disorders	Flat affect	15
809	Psychiatric disorders	Stress	15
810	Psychiatric disorders	Dyssomnia	15
811	Psychiatric disorders	Panic attack	15
812	Psychiatric disorders	Depressed mood	15
813	Psychiatric disorders	Dysphemia	15
814	Psychiatric disorders	Sleep disorder	15
815	Psychiatric disorders	Aggression	15
816	Psychiatric disorders	Restlessness	15
817	Psychiatric disorders	Affect lability	15
818	Psychiatric disorders	Acute psychosis	15
819	Psychiatric disorders	Mental disorder	15
820	Psychiatric disorders	Claustrophobia	15
821	Psychiatric disorders	Dysphoria	15
822	Psychiatric disorders	Tension	15
823	Psychiatric disorders	Initial insomnia	7.5

824	Psychiatric disorders	Agitation	7.5
825	Psychiatric disorders	Depersonalisation	15
826	Psychiatric disorders	Derealisation	15
827	Psychiatric disorders	Loss of libido	10
828	Psychiatric disorders	Nervousness	10
829	Psychiatric disorders	Distractibility	10
830	Psychiatric disorders	Abnormal behaviour	10
831	Psychiatric disorders	Depressive symptom	15
832	Psychiatric disorders	Conversion disorder	15
833	Psychiatric disorders	Post-traumatic stress disorder	15
834	Psychiatric disorders	Anxiety disorder	10
835	Psychiatric disorders	Burnout syndrome	15
836	Psychiatric disorders	Dysthymic disorder	10
837	Psychiatric disorders	Emotional distress	10
838	Psychiatric disorders	Phobia	10
839	Psychiatric disorders	Affective disorder	10
840	Psychiatric disorders	Confusional state	15
841	Psychiatric disorders	Neurosis	15
842	Psychiatric disorders	Disturbance in sexual arousal	10
843	Psychiatric disorders	Daydreaming	10
844	Psychiatric disorders	Abnormal dreams	10
845	Psychiatric disorders	Psychotic disorder	15
846	Psychiatric disorders	Mood swings	10
847	Psychiatric disorders	Alcohol abuse	15

848	Psychiatric disorders	Emotional disorder	10
849	Renal and urinary disorders	Pollakiuria	10
850	Renal and urinary disorders	Urinary incontinence	15
851	Renal and urinary disorders	Nocturia	10
852	Renal and urinary disorders	Residual urine	10
853	Renal and urinary disorders	Micturition urgency	10
854	Renal and urinary disorders	Dysuria	10
855	Renal and urinary disorders	Urinary retention	10
856	Renal and urinary disorders	Glycosuria	10
857	Renal and urinary disorders	Haematuria	10
858	Renal and urinary disorders	Leukocyturia	10
859	Renal and urinary disorders	Urge incontinence	10
860	Renal and urinary disorders	Urinary hesitation	10
861	Renal and urinary disorders	Renal pain	10
862	Renal and urinary disorders	Calculus urinary	15
863	Renal and urinary disorders	Bladder pain	10
864	Renal and urinary disorders	Urinary tract disorder	10
865	Renal and urinary disorders	Renal colic	15
866	Renal and urinary disorders	Polyuria	10
867	Renal and urinary disorders	Nephrolithiasis	10
868	Renal and urinary disorders	Renal cyst	10
869	Renal and urinary disorders	Neurogenic bladder	10
870	Renal and urinary disorders	Urine odour abnormal	7.5
871	Renal and urinary disorders	Chromaturia	7.5

872	Renal and urinary disorders	Urinary tract obstruction	15
873	Renal and urinary disorders	Urine abnormality	10
874	Renal and urinary disorders	Bladder spasm	10
875	Reproductive system and breast disorders	Menstruation irregular	10
876	Reproductive system and breast disorders	Erectile dysfunction	10
877	Reproductive system and breast disorders	Postmenopausal haemorrhage	10
878	Reproductive system and breast disorders	Amenorrhoea	10
879	Reproductive system and breast disorders	Vaginal discharge	10
880	Reproductive system and breast disorders	Metrorrhagia	10
881	Reproductive system and breast disorders	Ovarian cyst	10
882	Reproductive system and breast disorders	Varicose veins vaginal	10
883	Reproductive system and breast disorders	Menorrhagia	10
884	Reproductive system and breast disorders	Menstrual disorder	10
885	Reproductive system and breast disorders	Dysmenorrhoea	10
886	Reproductive system and breast disorders	Polymenorrhoea	10
887	Reproductive system and breast disorders	Pelvic pain	10
888	Reproductive system and breast disorders	Vaginal haemorrhage	10
889	Reproductive system and breast disorders	Hypomenorrhoea	10
890	Reproductive system and breast disorders	Cervical dysplasia	15

891	Reproductive system and breast disorders	Breast pain	10
892	Reproductive system and breast disorders	Vulvovaginal dryness	10
893	Reproductive system and breast disorders	Menometrorrhagia	10
894	Reproductive system and breast disorders	Genital discharge	10
895	Reproductive system and breast disorders	Benign prostatic hyperplasia	10
896	Reproductive system and breast disorders	Endometrial hyperplasia	15
897	Reproductive system and breast disorders	Endometrial hypertrophy	10
898	Reproductive system and breast disorders	Menopausal symptoms	10
899	Reproductive system and breast disorders	Uterine polyp	10
900	Reproductive system and breast disorders	Gynaecomastia	10
901	Reproductive system and breast disorders	Sexual dysfunction	10
902	Reproductive system and breast disorders	Breast disorder	10
903	Reproductive system and breast disorders	Cervical cyst	10
904	Reproductive system and breast disorders	Fibrocystic breast disease	10
905	Reproductive system and breast disorders	Uterine cervical erosion	10
906	Reproductive system and breast disorders	Galactocele	10
907	Reproductive system and breast disorders	Adenomyosis	15
908	Reproductive system and breast disorders	Ovarian disorder	15

909	Reproductive system and breast disorders	Breast tenderness	10
910	Reproductive system and breast disorders	Vaginal disorder	10
911	Reproductive system and breast disorders	Premenstrual syndrome	10
912	Reproductive system and breast disorders	Prostatitis	10
913	Reproductive system and breast disorders	Endometriosis	15
914	Respiratory, thoracic and mediastinal disorders	Cough	7.5
915	Respiratory, thoracic and mediastinal disorders	Epistaxis	7.5
916	Respiratory, thoracic and mediastinal disorders	Oropharyngeal pain	7.5
917	Respiratory, thoracic and mediastinal disorders	Dyspnoea	7.5
918	Respiratory, thoracic and mediastinal disorders	Nasal congestion	7.5
919	Respiratory, thoracic and mediastinal disorders	Pulmonary embolism	20
920	Respiratory, thoracic and mediastinal disorders	Asthma	15
921	Respiratory, thoracic and mediastinal disorders	Chronic obstructive pulmonary disease	20
922	Respiratory, thoracic and mediastinal disorders	Rhinitis allergic	10
923	Respiratory, thoracic and mediastinal disorders	Dysphonia	7.5
924	Respiratory, thoracic and mediastinal disorders	Dyspnoea exertional	15
925	Respiratory, thoracic and mediastinal disorders	Vocal cord inflammation	7.5
926	Respiratory, thoracic and mediastinal disorders	Rhinorrhoea	7.5

927	Respiratory, thoracic and mediastinal disorders	Nasal dryness	7.5
928	Respiratory, thoracic and mediastinal disorders	Pleurisy	20
929	Respiratory, thoracic and mediastinal disorders	Pharyngeal inflammation	7.5
930	Respiratory, thoracic and mediastinal disorders	Pneumonia aspiration	20
931	Respiratory, thoracic and mediastinal disorders	Pulmonary oedema	20
932	Respiratory, thoracic and mediastinal disorders	Vocal cord disorder	7.5
933	Respiratory, thoracic and mediastinal disorders	Lung disorder	20
934	Respiratory, thoracic and mediastinal disorders	Bronchial obstruction	20
935	Respiratory, thoracic and mediastinal disorders	Productive cough	7.5
936	Respiratory, thoracic and mediastinal disorders	Interstitial lung disease	7.5
937	Respiratory, thoracic and mediastinal disorders	Obstructive airways disorder	20
938	Respiratory, thoracic and mediastinal disorders	Hyperventilation	15
939	Respiratory, thoracic and mediastinal disorders	Oropharyngeal blistering	15
940	Respiratory, thoracic and mediastinal disorders	Nasal mucosal disorder	7.5
941	Respiratory, thoracic and mediastinal disorders	Increased upper airway secretion	7.5
942	Respiratory, thoracic and mediastinal disorders	Snoring	7.5
943	Respiratory, thoracic and mediastinal disorders	Wheezing	7.5
944	Respiratory, thoracic and mediastinal disorders	Pharyngeal erythema	10

945	Respiratory, thoracic and mediastinal disorders	Pharyngeal oedema	10
946	Respiratory, thoracic and mediastinal disorders	Sleep apnoea syndrome	15
947	Respiratory, thoracic and mediastinal disorders	Emphysema	20
948	Respiratory, thoracic and mediastinal disorders	Pulmonary fibrosis	20
949	Respiratory, thoracic and mediastinal disorders	Respiratory gas exchange disorder	20
950	Respiratory, thoracic and mediastinal disorders	Bronchitis chronic	20
951	Respiratory, thoracic and mediastinal disorders	Haemoptysis	10
952	Respiratory, thoracic and mediastinal disorders	Respiratory disorder	10
953	Respiratory, thoracic and mediastinal disorders	Lung cyst	10
954	Respiratory, thoracic and mediastinal disorders	Orthopnoea	10
955	Respiratory, thoracic and mediastinal disorders	Sinus congestion	10
956	Respiratory, thoracic and mediastinal disorders	Bronchial wall thickening	10
957	Respiratory, thoracic and mediastinal disorders	Throat tightness	10
958	Respiratory, thoracic and mediastinal disorders	Hiccups	7.5
959	Respiratory, thoracic and mediastinal disorders	Dry throat	7.5
960	Respiratory, thoracic and mediastinal disorders	Pleuritic pain	15
961	Respiratory, thoracic and mediastinal disorders	Pharyngeal ulceration	15
962	Respiratory, thoracic and mediastinal disorders	Respiratory tract congestion	10

963	Respiratory, thoracic and mediastinal disorders	Tonsillar hypertrophy	7.5
964	Respiratory, thoracic and mediastinal disorders	Pulmonary artery wall hypertrophy	20
965	Respiratory, thoracic and mediastinal disorders	Postnasal drip	7.5
966	Respiratory, thoracic and mediastinal disorders	Pleural fibrosis	20
967	Respiratory, thoracic and mediastinal disorders	Sneezing	7.5
968	Skin and subcutaneous tissue disorders	Eczema	7.5
969	Skin and subcutaneous tissue disorders	Seborrhoeic dermatitis	7.5
970	Skin and subcutaneous tissue disorders	Dermatitis	7.5
971	Skin and subcutaneous tissue disorders	Rash	7.5
972	Skin and subcutaneous tissue disorders	Acne	7.5
973	Skin and subcutaneous tissue disorders	Actinic keratosis	7.5
974	Skin and subcutaneous tissue disorders	Dry skin	7.5
975	Skin and subcutaneous tissue disorders	Lentigo	7.5
976	Skin and subcutaneous tissue disorders	Pityriasis rosea	7.5
977	Skin and subcutaneous tissue disorders	Hyperhidrosis	7.5
978	Skin and subcutaneous tissue disorders	Acrodermatitis	7.5
979	Skin and subcutaneous tissue disorders	Hyperkeratosis palmaris and plantaris	7.5
980	Skin and subcutaneous tissue disorders	Purpura	7.5
981	Skin and subcutaneous tissue disorders	Dermatitis allergic	7.5
982	Skin and subcutaneous tissue disorders	Erythema	7.5
983	Skin and subcutaneous tissue disorders	Dermatitis atopic	7.5
984	Skin and subcutaneous tissue disorders	Hypoaesthesia facial	7.5

985	Skin and subcutaneous tissue disorders	Seborrhoea	7.5
986	Skin and subcutaneous tissue disorders	Pruritus	7.5
987	Skin and subcutaneous tissue disorders	Skin ulcer	7.5
988	Skin and subcutaneous tissue disorders	Rosacea	7.5
989	Skin and subcutaneous tissue disorders	Drug eruption	7.5
990	Skin and subcutaneous tissue disorders	Lividity	7.5
991	Skin and subcutaneous tissue disorders	Urticaria	7.5
992	Skin and subcutaneous tissue disorders	Alopecia	7.5
993	Skin and subcutaneous tissue disorders	Swelling face	7.5
994	Skin and subcutaneous tissue disorders	Rash pruritic	7.5
995	Skin and subcutaneous tissue disorders	Rash maculo-papular	7.5
996	Skin and subcutaneous tissue disorders	Skin disorder	7.5
997	Skin and subcutaneous tissue disorders	Onychoclasis	7.5
998	Skin and subcutaneous tissue disorders	Photosensitivity reaction	7.5
999	Skin and subcutaneous tissue disorders	Dermatitis contact	7.5
1000	Skin and subcutaneous tissue disorders	Neurodermatitis	7.5
1001	Skin and subcutaneous tissue disorders	Nail bed inflammation	7.5
1002	Skin and subcutaneous tissue disorders	Blood blister	7.5
1003	Skin and subcutaneous tissue disorders	Psoriasis	7.5
1004	Skin and subcutaneous tissue disorders	Granuloma annulare	7.5
1005	Skin and subcutaneous tissue disorders	Rash macular	7.5
1006	Skin and subcutaneous tissue disorders	Skin plaque	7.5
1007	Skin and subcutaneous tissue disorders	Skin hyperpigmentation	7.5
1008	Skin and subcutaneous tissue disorders	Skin reaction	7.5

1009	Skin and subcutaneous tissue disorders	Rash erythematous	7.5
1010	Skin and subcutaneous tissue disorders	Ecchymosis	7.5
1011	Skin and subcutaneous tissue disorders	Dyshidrosis	7.5
1012	Skin and subcutaneous tissue disorders	Erythema nodosum	7.5
1013	Skin and subcutaneous tissue disorders	Dermatitis bullous	7.5
1014	Skin and subcutaneous tissue disorders	Telangiectasia	7.5
1015	Skin and subcutaneous tissue disorders	Hyperkeratosis	7.5
1016	Skin and subcutaneous tissue disorders	Alopecia universalis	7.5
1017	Skin and subcutaneous tissue disorders	Henoch-Schonlein purpura	7.5
1018	Skin and subcutaneous tissue disorders	Erythema multiforme	7.5
1019	Skin and subcutaneous tissue disorders	Spider naevus	7.5
1020	Skin and subcutaneous tissue disorders	Skin lesion	7.5
1021	Skin and subcutaneous tissue disorders	Ingrowing nail	7.5
1022	Skin and subcutaneous tissue disorders	Palmar erythema	7.5
1023	Skin and subcutaneous tissue disorders	Skin irritation	7.5
1024	Skin and subcutaneous tissue disorders	Xeroderma	7.5
1025	Skin and subcutaneous tissue disorders	Keloid scar	7.5
1026	Skin and subcutaneous tissue disorders	Skin exfoliation	7.5
1027	Skin and subcutaneous tissue disorders	Night sweats	7.5
1028	Skin and subcutaneous tissue disorders	Onychomadesis	7.5
1029	Skin and subcutaneous tissue disorders	Skin discolouration	7.5
1030	Skin and subcutaneous tissue disorders	Pruritus generalised	7.5
1031	Skin and subcutaneous tissue disorders	Skin depigmentation	7.5
1032	Skin and subcutaneous tissue disorders	Dandruff	7.5

1033	Skin and subcutaneous tissue disorders	Blister	7.5
1034	Skin and subcutaneous tissue disorders	Precancerous skin lesion	7.5
1035	Skin and subcutaneous tissue disorders	Scar	7.5
1036	Skin and subcutaneous tissue disorders	Petechiae	7.5
1037	Skin and subcutaneous tissue disorders	Campbell de Morgan spots	7.5
1038	Skin and subcutaneous tissue disorders	Keratosis pilaris	7.5
1039	Skin and subcutaneous tissue disorders	Skin striae	7.5
1040	Skin and subcutaneous tissue disorders	Skin warm	7.5
1041	Skin and subcutaneous tissue disorders	Pityriasis	7.5
1042	Skin and subcutaneous tissue disorders	Dermal cyst	7.5
1043	Skin and subcutaneous tissue disorders	Bromoderma	7.5
1044	Skin and subcutaneous tissue disorders	Haemorrhage subcutaneous	7.5
1045	Skin and subcutaneous tissue disorders	Cold sweat	7.5
1046	Skin and subcutaneous tissue disorders	Increased tendency to bruise	7.5
1047	Skin and subcutaneous tissue disorders	Rash papular	7.5
1048	Skin and subcutaneous tissue disorders	Hirsutism	7.5
1049	Skin and subcutaneous tissue disorders	Pigmentation disorder	7.5
1050	Skin and subcutaneous tissue disorders	Skin hypopigmentation	7.5
1051	Skin and subcutaneous tissue disorders	Eczema nummular	7.5
1052	Skin and subcutaneous tissue disorders	Skin hypertrophy	7.5
1053	Skin and subcutaneous tissue disorders	Pityriasis alba	7.5
1054	Skin and subcutaneous tissue disorders	Chloasma	7.5
1055	Surgical and medical procedures	Uterine operation	20
1056	Vascular disorders	Varicose vein	7.5

1057	Vascular disorders	Thrombophlebitis superficial	15
1058	Vascular disorders	Phlebitis	15
1059	Vascular disorders	Hypertension	10
1060	Vascular disorders	Venous insufficiency	10
1061	Vascular disorders	Circulatory collapse	10
1062	Vascular disorders	Lymphoedema	10
1063	Vascular disorders	Haematoma	10
1064	Vascular disorders	Hypotension	10
1065	Vascular disorders	Peripheral coldness	10
1066	Vascular disorders	Vein disorder	10
1067	Vascular disorders	Hot flush	10
1068	Vascular disorders	Flushing	10
1069	Vascular disorders	Labile hypertension	10
1070	Vascular disorders	Vascular insufficiency	10
1071	Vascular disorders	Diastolic hypertension	10
1072	Vascular disorders	Poor peripheral circulation	10
1073	Vascular disorders	Raynaud's phenomenon	10
1074	Vascular disorders	Blood pressure fluctuation	10
1075	Vascular disorders	Aortic dilatation	15

[1] Superior Term (MedDRA definition);
[2] Preferred Term (MedDRA definition);
